# Chronic debilitation in stranded loggerhead sea turtles (*Caretta caretta*) in the southeastern United States: Morphometrics and clinicopathological findings

**DOI:** 10.1371/journal.pone.0200355

**Published:** 2018-07-10

**Authors:** Nicole I. Stacy, Jennifer M. Lynch, Michael D. Arendt, Larisa Avens, Joanne Braun McNeill, Carolyn Cray, Rusty D. Day, Craig A. Harms, A. Michelle Lee, Margie M. Peden-Adams, Kelly Thorvalson, Al L. Segars, Terry M. Norton

**Affiliations:** 1 Department of Comparative, Diagnostic, and Population Medicine, College of Veterinary Medicine, University of Florida, Gainesville, FL, United States of America; 2 National Institute of Standards and Technology, Chemical Sciences Division, Hollings Marine Laboratory, Charleston, SC, United States of America; 3 South Carolina Department of Natural Resources, Charleston, SC, United States of America; 4 NOAA, National Marine Fisheries Service, Beaufort Laboratory, Beaufort, NC, United States of America; 5 Division of Comparative Pathology, Department of Pathology & Laboratory Medicine, University of Miami School of Medicine, Miami, FL, United States of America; 6 North Carolina State University, Center for Marine Science and Technology, Morehead City, NC, United States of America; 7 Medical University of South Carolina, Charleston, SC, United States of America; 8 South Carolina Aquarium, Charleston, SC, United States of America; 9 Georgia Sea Turtle Center, Jekyll Island Authority, GA, United States of America; 10 St. Catherines Island Foundation, Midway, GA, United States of America; University of Bari, ITALY

## Abstract

Chronically debilitated loggerhead sea turtles (*Caretta caretta*) (DT) are characterized by emaciation, lethargy, and heavy barnacle coverage. Although histopathological findings associated with this condition have been reported, only limited data is available on health variables with clinical application. The objectives of this study were to 1) to compare morphometrics, clinicopathological variables, and immune functions of DTs to a group of apparently healthy loggerhead turtles to better understand the pathophysiology of the condition and 2) to assess health parameters in live debilitated turtles as they recovered during rehabilitation in order to identify potential prognostic indicators. We examined and sampled 43 DTs stranded from North Carolina to Florida for 47 health variables using standardized protocols to further characterize the condition. DTs were grouped into categories of severity of the condition, and those that survived were sampled at four time points through rehabilitation. All groups and time points were compared among DTs and to clinically healthy loggerhead turtles. Compared to healthy turtles, DTs had significantly lower body condition index, packed cell volume (PCV), total white blood cell (WBC) count, lymphocytes, glucose (Glc), total protein, all protein fractions as determined by electrophoresis, calcium (Ca), phosphorus (P), Ca:P ratio, potassium (K), lymphocyte proliferation, and greater heterophil toxicity and left-shifting, uric acid (UA), aspartate aminotransferase, creatine kinase, lysozyme, and respiratory burst. From admission to recovery, hematology and plasma chemistry data improved as expected. The most informative prognostic indicators, as determined by correlations with a novel severity indicator (based on survival times), were plastron concavity, P, albumin, total solids, UA, lymphocyte proliferation, WBC, K, Glc, Ca:P, and PCV. The results of this study document the wide range and extent of morphometric and metabolic derangements in chronically debilitated turtles. Monitoring morphometrics and clinicopathological variables of these animals is essential for diagnosis, treatment, and prognosis during rehabilitation.

## Introduction

The loggerhead sea turtle (*Caretta caretta*) distinct population segment inhabiting the Northwest Atlantic Ocean is listed as threatened on the U.S. Endangered Species Act [1]. This population nests predominantly on Florida’s Atlantic beaches, circumnavigates the Atlantic Ocean as young pelagic juveniles, and recruits back to U.S. coastal areas as older benthic-foraging juveniles. Benthic juvenile and adult loggerhead turtles are commonly observed in the stranding records along the southeast U.S. coast. Many of those found live are transported to rehabilitation centers where large resources of time and money are used for medical treatment with the objective to release them after successful recovery. Examination of stranded sea turtles can provide important information for understanding disease and stressors on sea turtle populations [2, 3]. Common causes of stranding in sea turtles worldwide include watercraft or other traumatic injuries, drowning from fisheries interactions, hypothermia in winter months, and disease (i.e., [2, 4–8]).

For decades, chronically debilitated loggerhead turtles have been documented in the sea turtle stranding record along the southeast U.S. Informally called “barnacle bills,” these debilitated turtles (DTs) present with emaciation, presence of numerous small barnacles covering the skin, and lethargy. Heavy epibiota can be normal on the carapace of healthy loggerhead turtles [8, 9], but they typically have only very low numbers of these commensals on the skin. Although specific cause(s) of chronic debilitation remain unknown, the current hypothesis is that this condition is consistent with the end stage of starvation and any cause preventing nutrient uptake or absorption can lead to this stage [10]. Comprehensive health assessments, including physical examinations, detailed necropsies with histopathology, hematology, plasma chemistry panels, and other diagnostics can help elucidate any commonality and may point towards potential cause(s). Published studies on clinicopathological data of debilitated turtles are very limited to date. Deem et al. [8] examined health indicators in stranded versus foraging and nesting loggerhead turtles from Georgia. The stranded group, which included a mixture of DTs and turtles with various injuries (i.e., trauma, hook, neurologic abnormalities), had lower packed cell volume (PCV), total protein (TP), total solids (TS), monocytes, glucose (Glc), potassium (K), blood urea nitrogen (BUN), albumin (ALB), and globulins (GLOB); and higher lymphocytes, total white blood cells (WBC), creatine kinase (CK), and uric acid (UA) compared to foraging loggerhead turtles. These health indicators are consistent with anemia, reduced food intake, muscle injury, and wasting in the stranded turtle group. Similarly, stranded loggerhead turtles with a number of different stranding conditions in the Canary Islands were assessed for hematology and plasma chemistry [11]. The malnourished group (7 of 95 cases) in that study showed some similarities to the findings of Deem et al. [8]. None of these reports were solely focused on chronically debilitated turtles, and a comprehensive evaluation of stranded debilitated turtles based on sampling of a broad suite of health variables during rehabilitation has been missing in the literature. Evaluation of data from turtles admitted from the same stranding cause has the potential to contribute to understanding the pathophysiology and may thus be helpful in the medical management of affected sea turtles during rehabilitation.

A perceived increase in DT strandings along the southeast U.S. prompted a workshop on St. Catherine’s Island, Georgia, U.S.A in 2003 with experts of diverse specialties. The workshop resulted in a multi-organizational, multi-state (North Carolina, South Carolina, Georgia, and Florida) effort to conduct the current detailed investigation focused solely on DTs, both live and dead, using standardized collection protocols. Standardized protocols included a large number of measured variables: physical examination, morphometrics, genetic haplotype identification, epibiota identification, hematology, plasma biochemistry including testosterone for sexing, immune function, blood cultures, fecal parasitology, organic and inorganic contaminant tissue sampling, necropsy with parasite sampling and multi-organ tissue sampling for histopathology. Turtles were sampled through rehabilitation at four specified time points.

The objectives of this study were to 1) to compare morphometrics, clinicopathological variables, and immune functions of DTs to a group of apparently healthy loggerhead turtles to better understand the pathophysiology of the condition and 2) to assess health parameters in live debilitated turtles as they recovered during rehabilitation in order to identify potential prognostic indicators.

## Materials and methods

### Debilitated and healthy turtles sampled

Required federal and state permits were obtained for all activities in this study, including blood sampling. Permit numbers were National Marine Fisheries Service permits 1245, 1260, 1540, and 1551; South Carolina Department of Natural Resources MTP-2013-0005, Georgia Department of Natural Resources 1141, North Carolina Wildlife Resources Commission 01ST50, 02ST54, and 07ST53, and Florida Florida Fish and Wildlife Conservation Commission MTP12-021, MTP 140, and MTP 163. Chronically debilitated loggerhead turtles (DTs) sampled for this study stranded from July 2001 through June 2007 along the coast of North Carolina, South Carolina, Georgia, and Florida, U.S.A. Seventeen were found dead; 25 were found alive. Depending on the stranding location, live DTs were transported to rehabilitation facilities, including the Karen Beasley Sea Turtle Rescue and Rehabilitation Center, Topsail Island, North Carolina; South Carolina Aquarium, Charleston, South Carolina; Georgia Department of Natural Resources, Brunswick, Georgia and St. Catherines Island, Georgia; Volusia Marine Science Center, Ponce Inlet, Florida; Mote Marine Laboratory, Sarasota, Florida; and The Living Seas, Orlando, Florida. Dead chronically debilitated loggerhead sea turtles were necropsied at various locations; the necropsy data will be presented in a forthcoming publication.

Over 300 free-ranging, live captured, apparently healthy loggerhead turtles (HTs) of similar size, region, and collection years as DTs were selected as control turtles. Not all variables were measured on each HT; sample sizes for each analysis are reported in the Materials and Methods section or in tables or Figs. HTs were captured by two long-term projects that maintain comprehensive databases of results. One project captured HTs in pound nets in Core Sound, North Carolina, through the NOAA National Marine Fisheries Service, Beaufort Laboratory. The second captured HTs in scientifically deployed trawl nets in coastal waters of South Carolina, Georgia, and northeastern Florida operated by the South Carolina Department of Natural Resources (SC DNR). All turtles captured by these two projects were measured and evaluated for abnormalities, such as emaciation, lethargy, injuries, and rehabilitation need. Abnormal findings are documented in the comments field of the databases. Turtles without comments related to debilitation or other abnormalities were selected as HTs. Life stage was determined based on SCL. As size at maturity is variable and some loggerheads begin to nest around 75 cm SCL [12, 13], 80 cm SCL was selected as the cut off between neritic juveniles and adults.

### Physical examination and morphometrics

Each stranded DT included in this study was defined as a chronically debilitated turtle by visual observation of the following criteria: extensive epibiota coverage on the skin, emaciation based on sunken appearance in axillary, inguinal, plastron regions or eyes (S1 Fig), lethargy, and no evidence of recent traumatic injury. Standardized digital photographs and measurements were taken, including straight carapace length (SCL) from the nuchal notch to the most posterior marginal notch, body depth, and body mass (S1 File). A body condition index was calculated as (mass in kg) divided by (cubed SCL in cm) and multiplied by 100,000 [14]. A novel measurement of plastron concavity was also taken by resting a straight edge across the widest point of the plastron and measuring the distance from the bottom of the straight edge to the surface of the midpoint of the sunken plastron (S1 File). After completion of this study, veterinarians have concluded that plastron measurements using this method should not be performed on live DTs so as not to cause cardiac tears from sharp or broken plastron bones. Live DTs were further examined by veterinarians upon admission to rehabilitation facilities, where they received medical treatment based on the judgment of the responsible veterinarian.

### DT health categories and sample collection time-points

Each DT was assigned to one of three health categories based on the individual’s status at admission or their outcome (Table 1). “Dead DTs” (n = 18) were sampled post-mortem because they were found dead or died shortly after stranding either naturally or by euthanasia. Blood was collected from three dead DTs from the heart or dorsal cervical sinus within a maximum of four hours after the last eye reflex was observed. “A-died” (n = 13) turtles were sampled alive at time of stranding and were destined for a rehabilitation center but subsequently died. “A-survived” (n = 12) turtles successfully recovered in rehabilitation and were released. All samples collected from “A-survived” and “A-died” turtles were collected before or at time of admission to rehabilitation facilities before administration of any medications. During rehabilitation of “A-survived” turtles, additional sample collection time points were defined as the following: “B” = approximately one week after the sea turtle started eating independently; “C” = approximately one to ten weeks after the “B” sample, after minor improvements in body mass, body condition, behaviors, or clinical data, but before rehabilitation staff considered the turtle clinically recovered; and “D” = immediately prior to release (Table 1). It was not possible to collect all B, C, or D samples from all admitted DTs because of logistical constraints. Veterinary care and release criteria were determined by staff at the various rehabilitation centers.

**Table 1:** Sample sizes and characteristics of debilitated (DT) and healthy loggerhead turtles sampled in the current study.

Turtle Group	DT dead	DT died	DT survived				Healthy*
Time Point	N/A	A	A	B	C	D	N/A
Timing of sampling	Upon dead stranding or after death	Upon live admission	Upon live admission	1 week after feeding on own	1 to 10 weeks after B, minor improvements	Upon release	Upon capture
Sample Group	DT dead [X]	A—died [Y]	A -survived [A]	B	C	D	HT
N	18	13	12	≤7	≤6	≤9	>300
Stranding years	2004–2005	2002–2007	2001–2006				1998–2009
Location (%):							
NC	17	23	42				63
SC	44	54	33				26
GA	28	15	25				9
FL	11	8	0				2
SCL (n-n) (%):							
<80 cm	83	85	75				88
>80 cm	17	15	25				12
Severity Indicator (days):							
Mean ± SD	0	6.23 ± 8.1	352.4 ± 123				N/A
Median (Range)	0 (0–0)	4 (1–31)	385 (47–463)				
N [sig dif from]	17 [YA]	13 [XA]	12 [XY]				

*Location and straight carapace length notch to notch (SCL n-n) percentages for healthy turtles were calculated on a representative 215 turtles.

Statistically significant differences among DT categories are shown in brackets in the sample size row.

### Blood collection and handling

Blood sampling was part of a larger protocol that included collection of additional samples (e.g. parasitology, genetics) that will be presented in a forthcoming publication (S1 File). Standardized protocols were followed for DT blood sample collection, handling, processing, and shipping to different laboratories. After sterile surgical preparation of the blood collection site, blood was collected from the dorsal cervical sinus using a 21-gauge 1.5 inch double-ended Vacutainer needle directly into several Vacutainers tubes (Becton Dickinson, Franklin Lakes, New Jersey, U.S.A.) [15]. Several heparinized tubes were collected for hematology, plasma biochemistry, plasma protein electrophoresis, and immune function analysis at all four time points. Blood was also collected using sterile technique into bacterial blood culture medium (BBL® Septi-Chek™, Becton Dickinson, Franklin Lakes, New Jersey, U.S.A.) at time points “A” and “D”. The blood culture specimens were kept at room temperature and Vacutainer tubes cooled until processed. From one well-mixed heparinized whole blood tube, four blood films were prepared within 30 min, PCV was measured in-house by centrifuging capillary tubes at 12,000 × g for 5 min, and 0.3 mL of whole blood was transferred to a cryovial. The remainder of this and one other heparinized tube were centrifuged at 4,000 × g for 5 min. Plasma was transferred to several cryovials (Corning Incorporated, Corning, New York, U.S.A.) for plasma biochemistry (0.6 mL), bile acids and plasma electrophoresis (0.5 mL), biliverdin (0.2 mL), archiving (remainder), and in-house measurements of TS using a hand-held refractometer. The 0.6 mL plasma aliquot and 0.3 mL whole blood aliquot were shipped overnight on cold packs, with blood culture bottles well insulated away from cold packs, and processed by a commercial laboratory (Antech Diagnostic, Memphis, TN). The remainder of blood tubes and plasma aliquots and two blood films were transported same day or overnight on cold packs to Charleston, South Carolina, for processing immediately after arrival for immune function assays (Medical University of South Carolina) as well as sample archival and contaminant analysis at the National Institute of Standards and Technology (NIST) (mercury [16] and persistent organic pollutants [Lynch, personal communication]). Blood from HTs was collected and processed similar to DTs. Blood was drawn as quickly as possible after capture and generally processed within one to eight hours of collection for plasma chemistry and eight to 48 hours of collection for immune function assays.

### Hematology

Blood films from DTs and a subset of 37 HTs were stained with Wright-Giemsa and blindly evaluated by one boarded clinical pathologist for WBC estimate, WBC differential (200 cells), and evaluation of blood cell morphology [17]. The WBC estimate was performed by multiplying the average number of WBC in 10 microscope fields × the objective power squared [18]. The erythroid regenerative response in anemic turtles was quantified by reporting the number of immature polychromatophilic erythrocytes per 100 mature erythrocytes as percentage (red blood cell [RBC] polychromasia %). In addition, subjective scores for RBC polychromasia, heterophil toxicity, and heterophil left-shift were used to characterize the degree by absent (0), mild (1), moderate (2), or marked (3).

### Plasma chemistry panels and blood cultures

The following plasma biochemical analytes were measured at Antech Diagnostics for all DTs using a Beckman Olympus AU 5431 Chemistry Analyzer (Beckman Coulter Inc., Brea, California, U.S.A.): ALB (bromocresol green method), aspartate aminotransferase (AST), BUN, calcium (Ca), chloride (Cl), CK, GLOB, Glc, phosphorus (P), K, sodium (Na), TP (biuret method), and UA. Plasma from HTs were frozen at or below -70 ^o^C for typically five to ten days before analysis. Data from HTs were a mixture of unpublished data measured at Antech Diagnostics (Beckman Olympus AU 5431) or previously published data measured at North Carolina State University (Roche/Hitachi 912, Roche Diagnostics, Indianapolis, IN) as reported in Keller et al. [14]. Blood culture results were reported by Antech at 14 days of incubation for DTs. No blood cultures were available from HTs.

### Plasma protein electrophoresis, bile acids, and biliverdin

Plasma protein electrophoresis was performed at the University of Miami (Miami, FL) on all available DT plasma samples and 19 HTs using SPEP-II agarose gels and the Beckman paragon electrophoresis system (Beckman-Coulter Corporation, Brea, California, U.S.A.) with quantification of TP by biuret method (Kodak 750 X R, Ortho Clinical Diagnostics, Rochester, New York, U.S.A.). The gels were run as described previously [19]. The percentage of protein fractions was quantified by laser densitometry and then each fraction value was calculated by multiplying the percentage of the fraction by the TP concentration. Bile acid analysis was performed at the University of Miami on all available DT plasma samples (no HT samples were analyzed) using the commercially available radioimmunoassay kit from MP Biomedicals (Solon, OH) as per recommendations by the manufacturer. The limit of detection was 0.1 μmol/L. Biliverdin concentrations were measured at the University of Georgia (Infectious Diseases Laboratory, Athens, GA) as previously described [20] on all available plasma samples from DTs and 20 HTs.

### Immune function assays

Immediately upon arrival, a heparinized blood tube was centrifuged at 42 × g for 25 min to harvest peripheral blood leukocytes (PBLs) from the buffy coat for immune function assays at the Medical University of South Carolina. Mitogen-stimulated lymphocyte proliferation (LP) was measured on all available DTs as well as up to 77 HTs as previously described [21, 22]. Briefly, viable PBLs were exposed to phytohemagglutinin P (PHA or lectin from red kidney bean (*Phaseolus vulgaris*); Sigma, St. Louis, MO, cat #L9132) and concanavalin A (ConA from jack bean [*Canavalia ensiformis*]; Sigma cat #C5275) to stimulate the T-lymphocyte proliferation, lipopolysaccharide (LPS from *Escherichia coli* 0111:B4; Sigma cat #L2630) to stimulate B-lymphocyte proliferation, and phorbol 12,13-dibutyrate (PDB; Sigma cat #P1269) to stimulate both T- and B-lymphocyte proliferation. The uptake of ^3^H-thymidine (ICN Biomedical, Irvine, CA) was measured from cells harvested onto Unifilter plates (Packard, Meridian, CT) using a Packard Top Count-NXT scintillation counter. The stimulation index (SI) is counts per minute (cpm) of mitogen-stimulated cells divided by the cpm of unstimulated control (media only) cells. The different incubation conditions are presented in the results as “mitogen_final mitogen concentration in the wells (μg/mL)_days of incubation” (e.g., PHA_5_5 means PHA at 5 μg/mL for five days).

Superoxide production, a measure of respiratory burst and thus innate immunity, was determined using PBLs by assessing nitroblue tetrazolium (NBT) conversion for all available DTs and 14 HTs. Briefly, 100 μL aliquots of PBLs diluted to 5 x 10^6^ cells/mL in complete medium (RPMI-1640, 10% fetal bovine serum, 50 IU penicillin and 50 μg streptomycin) were dispensed into 96-well plates containing triplicate wells of 60 μL of Ca ionphore (CI; 6.5 μL/mL complete media solution made from a 1 mg/mL frozen stock in dimethyl sulfoxide [DMSO]), phorbol 12,13-dibutyrate (PDB; 3.1 μL/mL complete media solution made from a 1 mg/mL frozen stock in DMSO), or supplemented RPMI-1640 (unstimulated wells). To each well, 140 μg NBT (10 mg NBT in 56.8 mL of Hanks' Balanced Salt Solution with 2 mmol/L CaCl_2_) was added. Plates were incubated for 50 min at 30°C and 5% CO_2_, then centrifuged at 1500 rpm (377 × g) for 3 min, and the supernatant was removed. KOH (120 μL of 2N) and 140 μL DMSO were added to each well, mixed by pipette, and plates were assessed for absorbance at 620 nm with a spectrophotometer (SpectraCount; Packard, Meridian, CT). The SI is absorbance units (AU) of stimulated cells divided by AU of unstimulated cells.

Plasma lysozyme activity, a measure of innate immunity, was determined using a standard turbidity assay described in Keller et al. [21] for all available DTs and 67 HTs.

### Statistical analysis

**Fig 1:**
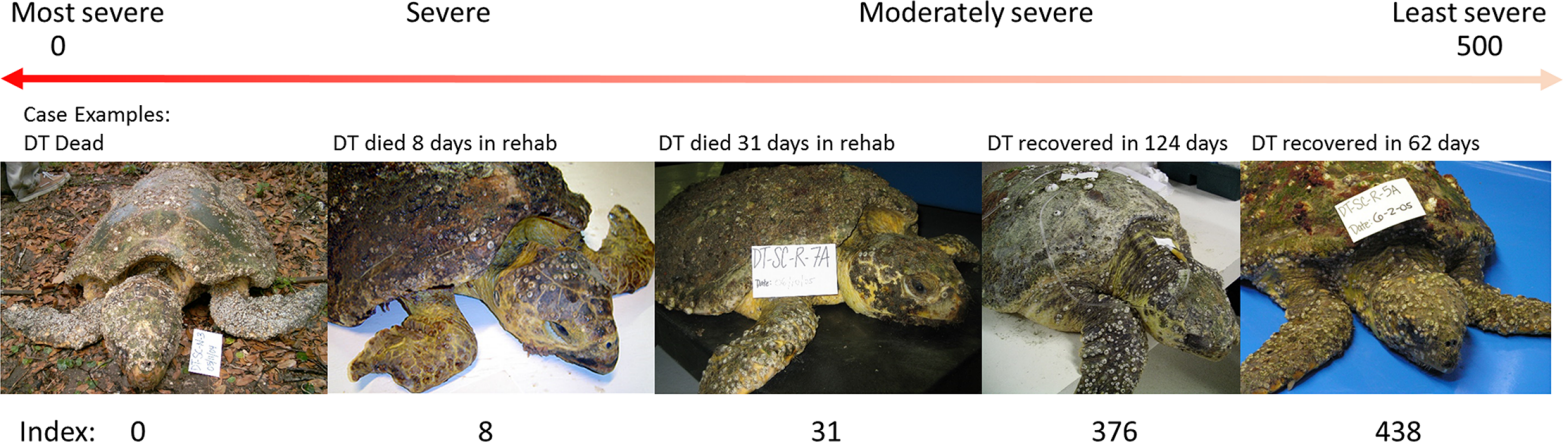
Diagram of severity indicator with five case examples of debilitated loggerhead sea turtles. Images show turtles at time of admission.

We developed a novel method to numerically and objectively represent the severity of debilitation for each turtle as a continuum from one discreet health category to another (Fig 1). We called this the severity indicator and used this continuous variable to assess correlations with clinicopathological variables to determine variables most predictive of prognosis. We considered the order of severity from worst to least as 1) DT dead, 2) A-died that died shortly after admission, 3) A-died that died several days into rehabilitation, 4) A-survived that recovered slowly, and 5) A-survived that recovered quickly (Fig 1). The duration of successful rehabilitation ranged from 47 days to 463 days, opportunistically providing an ideal large range for correlations. The severity indicator was calculated for A-survived turtles as the number of days between admission and a full recovery subtracted from 500 (an arbitrary value greater than the longest successful rehabilitation) so that the largest number represents the least severe turtle. For A-died turtles, the severity indicator was calculated as the number of days between the A sample and their death. Since those ranged from 1 to 31 days, again the larger the severity indicator, presumably the less severe the condition upon admission. DT dead were assigned the worst severity indicator of zero.

Statistical testing was performed using JMP 7.0.2 software (SAS, Cary, NC). Shapiro Wilk W and Bartlett tests were used to assess the distribution of the data and homoscedasticity, respectively. When either of these assumptions was not met, the data were log transformed. If data did not meet the assumptions, we chose the data (either raw or transformed) that were more normally distributed and more homoscedastic and checked statistical outcomes with non-parametric tests. To assess differences in health variables among A-survived, B, C, and D categories (e.g., improvement through rehabilitation), a repeated measures analysis of variance was used. If p<0.05, then paired t-tests were used to assess differences between each time point. Since these paired t-tests had to be performed individually (e.g. A-survived vs. B), a Bonferoni correction was used to determine significance (p<0.00883). To assess differences among all groups, including dead DTs, A-died, and HTs, which are independent turtles without repeated sampling, an analysis of variance was used including all health categories. If p<0.05, then a Tukey multiple comparison test was used to determine which health categories were different from these three independent health categories (e.g., do recovered turtles (D category) differ from HT?). Significance for the Tukey test was determined as p<0.05. Spearman correlations were performed for the severity indicator and all other health variables to investigate if more severely debilitated turtles had poorer health indicators and to determine which health variables were the most predictive of severity of the condition, thus providing an objective measure of prognosis. Values below the detection limit for bile acids were substituted with half the detection limit (0.05 μmol/L) for statistical testing. Because several samples had biliverdin concentrations below two different detection limits, statistical tests using the NADA package in R that appropriately handle left-censored data were used [23]. This also required a Bonferroni correction of alpha due to the 21 pairwise comparisons made (p<0.00238 was considered significant when using this test).

To investigate methodology differences in TP (biuret method) vs TS (refractometry) and ALB by bromocresol green method vs protein electrophoresis, Spearman correlations and paired t-tests were performed. Comparisons among TP, ALB, and TS included data only from DTs (all DT categories and time points were combined).

## Results and discussion

### Characteristics of sampled DTs

A total of 43 live and dead DTs from North Carolina, South Carolina, Georgia, and Florida were included in this study (Table 1). Of the turtles admitted alive (n = 25), 13 (52%) died. The “A-died” category included ten turtles that died within eight days after admission, one that died at day 31, and two that were euthanized on the day of admission. These data highlight the high mortality rate and guarded prognosis for DTs. The DTs of the category “A-survived” had an average rehabilitation time of 148 days (range 47 to 463 days). For live turtles during rehabilitation, samples were collected at time point “B” within 6 to 32 days after admission, “C” within 14 to 80 days after admission and “D” within 47 to 463 days after admission. In comparison, the turtles in the current study were in rehabilitation longer (mean = 148 days) than loggerhead turtles that stranded due to malnutrition in Gran Canaria Island, Spain (median = 43 days) [24]. This suggests that loggerheads in the Spanish study likely represent a less severe degree of emacation than compared to DTs of our study.

Most DTs sampled in this study stranded in SC (Table 1). This was an artifact of sampling effort being strongest in SC and should not be considered typical for spatial stranding trends of DTs in the southeast U.S. Approximately 80% of the DTs were categorized as neritic juveniles (<80 cm SCL) and 20% were adults. Seasonally, stranded DTs included in this study peaked between May and July (n = 4 in April, 17 in May, 9 in June, 6 in July, 3 in August, 0 in September, and 4 in October). Since season influences the immune system of reptiles [25], it is important to note that immunology was performed on HTs captured only in the summer months, coinciding with the peak season of the DT stranding.

**Fig 2:**
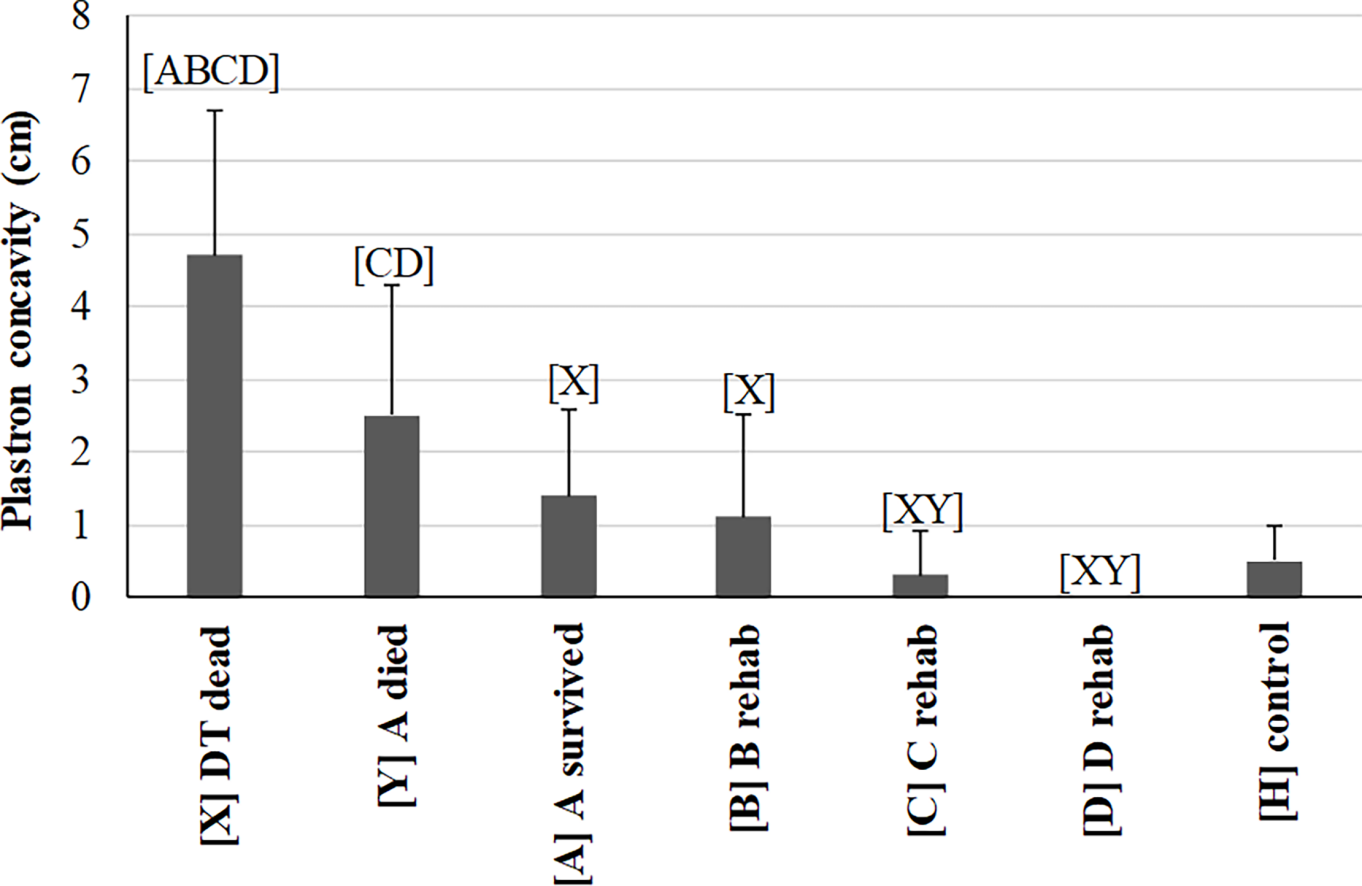
Comparison of plastron concavity measured in debilitated loggerhead sea turtles (DTs) before and during rehabilitation and compared to healthy loggerhead turtles (means and one standard deviation). Lines represent an individual turtle through rehabilitation. Each turtle group on the x-axis was significantly different (p<0.05 or p<0.00883 for repeated measures) from groups represented by letters above data. Sample size for each group: 15 [X], 9 [Y], 6 [A]–[D], 1[H].

The severity indicator (a measurement of the number of days to death or recovery) significantly decreased with increasing severity across the three DT categories as expected (Table 1). Morphometric data and significant differences among all seven health categories of turtles are summarized in S1 Table. SCL did not differ among the seven categories which suggested turtles of similar sizes and age classes were being compared. Body mass improved from time points A and B to time point D during rehabilitation as expected. Our novel method for quantitatively measuring plastron concavity (S1 File) proved to show expected trends (Fig 2): it was deepest in DT dead (deeper compared to all other categories) and A-died (deeper than C, D) with steady improvement through rehabilitation to a complete lack of concavity by the time of release. It is important to note that this measurement is not recommended in live DTs due to the high risk of cardiac tears from plastron bones when turning DTs onto their backs. Body condition index was lower in DT dead, A-died, and A-survived compared to D and HTs, whereas the index at time points B and C were lower only compared to HTs (S1 Table). These findings confirm the extent of emaciation that defines DTs during visual and physical examination. The significant improvements of body mass, plastron concavity, and body condition index by time point D during rehabilitation are consistent with the reversal from a catabolic to an anabolic state and restoration of energy stores. This is the first study to present these improvements specifically in debilitated loggerhead turtles.

### Blood culture

Blood cultures for 15 DTs (“A-survived” n = 6; “A-died” n = 7; “DT dead” n = 2) were collected at time of admission. Five samples were positive; one microorganism was found in each of the positive samples: *Aeromonas hydrophila/caviae* (A-survived), *Pseudomonas putida* (A-died*)*, *Vibrio parahemolyticus* (A-died), *Shewanella putrifaciens* (dead DT), *Shewanella algae* (dead DT). The *Shewanella sp*. cultured from the dead DTs could have resulted from post-mortem contamination, while the other organisms may have pathogenic significance in a clinically sick turtle. All blood cultures collected at time point D (n = 4) were negative, likely as the result from treatment and immune response.

### Hematology and morphological evaluation of blood films

Hematology results and significant differences among the seven turtle categories are summarized in S2 Table. Significant differences in one or more of three DT categories at the time of admission (Dead DT, A-died, or A-survived) compared to HTs included lower PCV, total WBC, heterophils, lymphocytes, and monocytes and higher degrees of heterophil toxicity and left-shifting. The lower PCV, indicating anemia, was consistent with stranded turtles from Georgia in Deem et al. [8] and the malnourished group from the Canary Islands in Casal and Oros [11]. This similarity across studies indicates that PCV is a clinically important variable when examining sick loggerhead turtles. Deem et al. [8] also found lower monocytes, but no change in total WBC or heterophils and an opposite trend (elevation) for lymphocytes. Casal and Oros [11] observed a similar decrease in total WBC and lymphocytes, but opposite trends for heterophils and monocytes. The differences among studies in cell counts could be due to different causes for stranding or emaciation, different stages of disease progression, or differences in methodology.

During rehabilitation, between A-survived and time point B, PCV, and lymphocytes decreased, and heterophil:lymphocyte ratio, and heterophil toxicity and left shifting increased (S2 Table). This indicates that during this initial stage of supportive care, these blood analytes get worse before they begin to improve. This is undoubtedly a very active phase in which supportive nutritional care and antimicrobial administration work together to mount significant and diverse physiological changes to reverse catabolism. Rehydration may cause PCV to decrease initially until hematopoietic tissues respond, notably to the anemia. Nutrition appears to provide the energy needed for hematopoietic and lymphoid tissues to mount effective immune responses.

**Fig 3:**
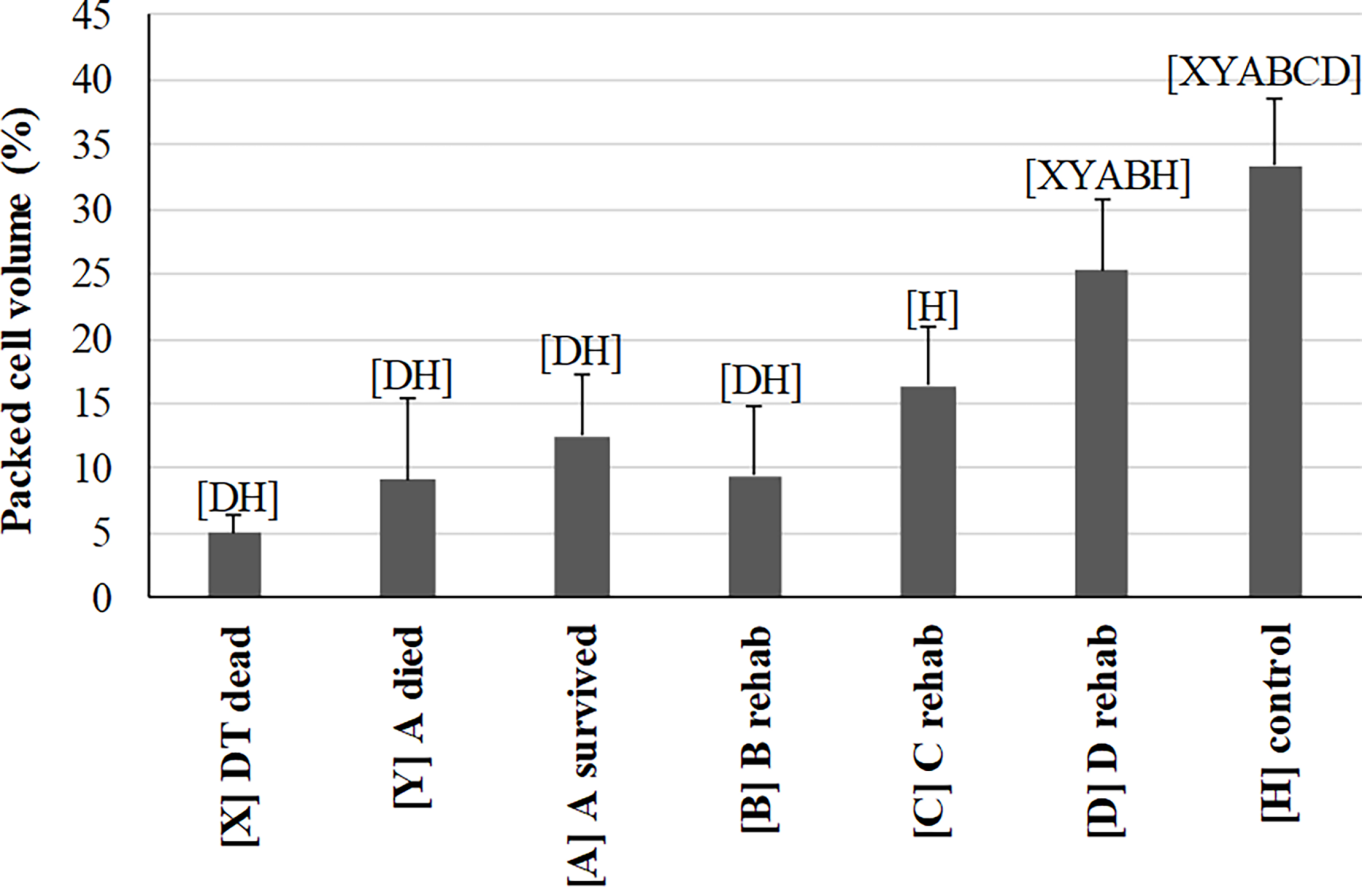
Comparison of packed cell volume (PCV) measured in debilitated loggerhead sea turtles (DTs) before and during rehabilitation and compared to healthy loggerhead turtles (means and one standard deviation). Lines represent an individual turtle through rehabilitation. Each turtle group on the x-axis was significantly different (p<0.05 or p<0.00883 for repeated measures) from groups represented by letters above data. Sample size for each group: 2 [X], 10 [Y], 11 [A], 7 [B], 6 [C], 9 [D], 69 [H].

PCV (Fig 3) was significantly and dramatically lower in DT dead, A-died, A-survived, and B compared to D and HT. During rehabilitation, PCV dropped at B likely in response to fluid therapy and rehydration, significantly improved by D, but never recovered to ranges of HTs. The only other hematological variable that significantly differed between A-survived and D was an increase in RBC polychromasia evaluated as by score and percentage (S2 Table). Eosinophils and basophils showed little to no differences between DTs and HTs or between A-survived and D. These findings are similar to Harms et al. [26], in which PCV increased and no change in WBC counts or the leukocyte differential counts were observed in loggerheads undergoing rehabilitation after stranding from various causes not including debilitation. No blood parasites or other infectious agents were observed on any blood film.

The morphological evaluation of RBC polychromasia is critical in the identification of erythroid regeneration in anemic reptiles. Given the lack of standardization in the quantification of the erythroid regenerative response in non-mammalian vertebrates, two approaches were attempted in this study. Both a subjective RBC polychromasia score and a numerical RBC polychromasia percentage provided similar results with concurrent improvement in PCV and a peak in RBC polychromasia by both methods at time point C (S2 Table). At stranding, eight out of 12 DTs had severe, non-regenerative anemia (PCV < 12%, RBC polychromasia score = zero). The remaining four DTs had moderate, non-regenerative anemia (PCV = 14% to 21%, RBC polychromasia score = zero). We investigated the time it took to observe the first evidence of erythroid regeneration in the DTs that were followed through rehabilitation using the criteria of PCV rising above 14% and RBC polychromasia scores becoming at least one. This was observed at 56 days after admission (mean; range 24–108 days) occurring in time points B or C. But it took longer before the PCV increased to a minimum of ≥ 20%, which occurred by day 103 (mean; range 36–148 days) at time points C or D for all turtles that were closely followed using complete sets of blood films for all time points. However, in some turtles, no overt erythroid regeneration (e.g., polychromasia) was observed at the defined time points during rehabilitation despite resolving anemia. Lack of an observed erythroid response by blood film evaluation may have been due to variable frequency of sampling during recovery. Had the study been extended, we may have observed a morphologically recognizable erythroid response. The clinical importance of the long duration to full recovery of PCV underlines the severity of the condition and the duration of supportive care needed in order to elicit a response by hematopoietic tissues to the chronic anemia.

The degree of the anemia and slow recovery of PCV in the admitted DTs reflects the severity of debilitation. The complexity of the disorder and end-stage presentation of the patients precludes identification of specific cause(s) of anemia; however, the causes are likely multifactorial and difficult if not impossible to identify as previously reported for a stranded group of loggerhead turtles [8]. Considerations for the causes of anemia in DTs include bone marrow suppression and reduced erythropoiesis from malnutrition, inflammatory and/or chronic disease(s), and/or bacterial or parasitic infections, among other ailments seen in the DTs. Regardless from which cause(s) the anemia initially resulted, the clinical significance of anemia lies in the reduced oxygen delivery to tissues, which may lead to further organ compromise from hypoxia, especially in highly vascularized tissues such as liver, kidneys, and lungs.

Leukocyte morphological changes can be useful prognostic indicators in mammals [27]. Mild to marked heterophil toxicity was observed in ten of 17 DTs at presentation or time of admission and heterophil left-shifting in 13 of these DTs. Low numbers of melanomacrophages (<5/blood film) were noted in two A-died, one A-survived and throughout its rehabilitation, and at the D time point for two additional A-survived turtles. Heterophil toxicity reached a score of zero by day 49 (mean; range 8–124; time points B or D), and heterophil left-shifting took longer to disappear with 95 days into rehabilitation (mean; range 56–151; time point D). The leukogram findings, especially the frequency and duration of heterophil left-shifting, indicate ongoing active systemic inflammation in the DTs. In mammals, neutrophil toxicity is considered non-specific and can be seen in various disorders, including systemic inflammation, bacterial or other infections, tissue necrosis, and some severe metabolic disorders [27]. In dogs, the presence of these cytoplasmic features of toxicity is associated with higher mortality rates, and their disappearance correlates well with clinical improvement, thus offering a prognostic indicator [27]. In reptiles, heterophil toxicity appears to have similar prognostic significance as well as heterophil left-shifting [17]. Left-shifting is defined as the presence of immature heterophils, which indicates a need for active production of these inflammatory cells by hematopoietic tissues in response to increased tissue demand. The data on these DTs support the usefulness of these morphological changes as diagnostic indicators and for monitoring. Moreover, melanomacrophages can be a rare finding in healthy reptiles, but an increased number circulating in ill reptilian patients may indicate chronic inflammation/disease, cachexia, or other nonspecific conditions [28, 29].

### Plasma chemistry

Mild hemolysis was present in one of three dead DTs, and plasma was green for three turtles sampled at admission (two A-survived and one A-died). Green plasma has been associated with starvation, liver disease, and hemolytic disease in sea turtles [29] and it is unknown how the green discoloration may affect the analysis of various chemistry analytes. Plasma biochemistry results and significant differences among the seven turtle categories are summarized in S3 Table. Severe metabolic derangements were observed in the three DT categories at the time of admission (Dead DT, A-died, or A-survived). Significant differences in one or more of those categories compared to HTs included decreased Glc, TP, all protein fractions, Ca, Ca:P ratio, and K and increased P, Na, Cl, UA, AST and CK. The reduced Glc and protein concentrations are consistent with other studies that examined stranded loggerhead turtles [8, 11, 30]. Deem et al. [8] also observed the decrease in K and increases in UA and CK in the stranded group, but not the changes we noted in other electrolytes or AST. The stranded turtles in the Canary Islands showed the opposite, lower UA and AST compared to the DTs in the current study [11]. These comparisons suggest that lack of nutrition (decreased Glc and TP) from anorexia is present in most stranded turtles, regardless of the cause of stranding, and the changes in electrolytes and AST seem more common in turtles with chronic debilitation.

**Fig 4:**
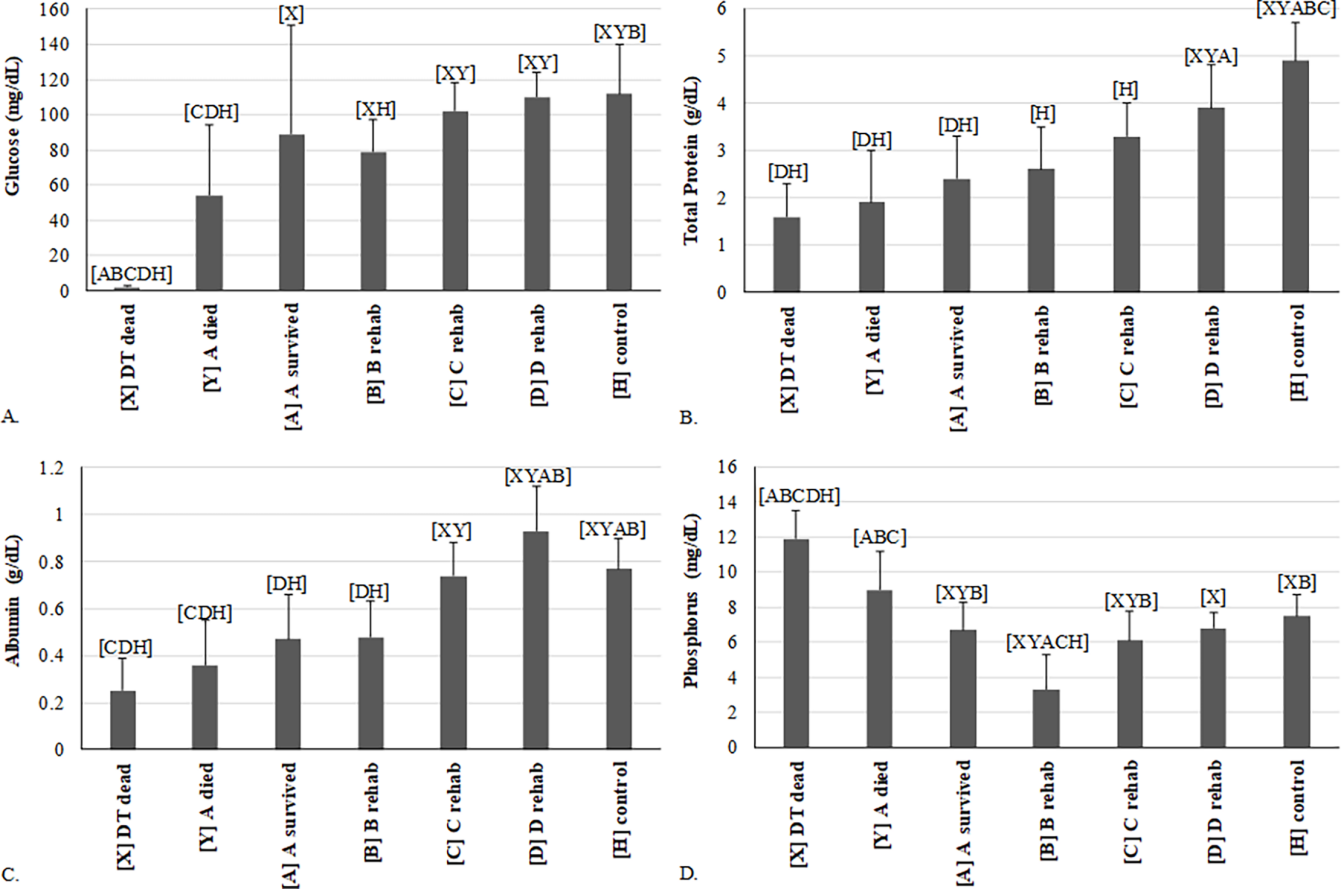
Comparison of variables measured in debilitated loggerhead sea turtles (DTs) before and during rehabilitation and compared to healthy loggerhead turtles (means and one standard deviation). Each turtle group on the x-axis was significantly different (p<0.05 or p<0.00883 for repeated measures) from groups represented by letters above data. (A) glucose; (B) total protein by biuret method at U. Miami; (C) albumin by plasma electrophoresis; (D) phosphorus. Lines represent an individual turtle through rehabilitation. For samples sizes in each diagram, please refer to S3 Table.

During rehabilitation, most variables improved as expected (S3 Table). Notable trends were observed for Glc, TP, ALB, and P (Fig 4). Interestingly, between A-survived and time point B, several variables, including Glc, Ca, K, AST, and CK, tended to worsen (S3 Table). This observation suggests that even with early treatment, plasma chemistry indicators of debilitation tend to worsen before improvement as critical physiologic changes occur during this initial phase reversing the effects of starvation. This mirrors the above discussed hematological finding suggestive of an inflammatory response after treatment initiation at time point B: the chemistry findings may indicate a stimulation of internal organs in response to supportive care in the effort to correct metabolic derangements after an extended time of anorexia and organ compromise. Other variables with statistically significant changes between A-survived and D include increases in alpha 1-, and beta-globulins, BUN, and K, and a decrease in AST (S3 Table). Variables that were not as severely deranged (e.g., showed little to no differences between DTs and HTs or between A-survived and D) were TS, ALB:GLOB ratio, bile acids, and biliverdin. Three other studies have followed plasma chemistry values through rehabilitation of loggerhead sea turtles [4, 26, 31]. The increase in BUN was consistently noted in all three studies, but none of the studies saw an increase in Glc, since only few turtles initially presented with hypoglycemia. The increases in TP, ALB, and GLOB were also observed in Harms et al. [26]. The increase in K was seen by Harms et al. [26] but not by Camacho et al. [31]. Harms et al. [26] observed an increase in P and no change in AST, which are different from the current study.

Glc, similar to PCV, dropped between A-survived and B with subsequent improvement by C to healthy ranges (Fig 4A). Hypoglycemia has been documented in stranded loggerhead turtles [8], especially those with malnutrition [11, 32]. Although the extremely low Glc concentrations in DT dead (≤2 mg/dL) may have been falsely decreased by prolonged contact of the plasma with erythrocytes *in vivo*, this artifact was likely mild, since all three blood samples were collected within four hours after death and immediately processed; additionally, this degree of severe hypoglycemia was also observed in a live DT upon admission. Based on our results, hypoglycemia in DTs may be associated with hyporexia or anorexia, exhaustion, exertion, malnutrition, decreased gluconeogenesis, and/or sepsis. One A-survived turtle had moderate hyperglycemia (244 mg/dL) at admission, which may be explained by metabolic stress or stress of stranding and/or handling in this individual. The Glc fell to 68 mg/dL 4 days after admission (B) in this individual turtle, and normalized by 41 and 62 days after admission (C and D, respectively) to between 115 and 111 mg/dL.

TP (Fig 4B) and ALB (Fig 4C) were lowest at admission of DTs but increased to ranges similar to HTs by D or C, respectively. All three groups of DTs were severely panhypoproteinemic at time of admission, with significant, steady improvement during rehabilitation and recovery to concentrations comparable to healthy turtles by time point D (S3 Table). Previously concluded causes for panhypoproteinemia in stranded loggerhead turtles include malnutrition, parasites, and protein-losing disorders [8]. The lower ALB: GLOB ratio in non-survivors is consistent with significantly lower ALB concentrations, with considerations including conditions with decreased ALB synthesis (e.g., inflammation, malabsorption/maldigestion, hepatic insufficiency) or albumin loss (e.g. through gastrointestinal tract, kidneys, skin, or blood loss). Pre-albumin, which includes thyroid-hormone-binding protein (transthyretin) and vitamin-binding proteins in other species [33, 34], as well as albumin, alpha-1-, alpha-2-, beta-, and gamma-globulins all demonstrated steady improvement during rehabilitation with ranges at recovery at or approaching that of HTs (S3 Table). Most protein fractions decreased between time points A and B, which tracks with PCV, and may be explained by rehydration and hemodynamic improvement from fluid treatment.

It was important to compare the different measures and methods of protein (TP, ALB, TS) to determine if methods substantially deviated from each other. The three investigated correlations were statistically significant (p<0.0001) with positive slopes, suggesting that generally each variable or method tracks the other, but they all fell below the 1:1 line: TP by biuret method [Antech] vs. TS (n = 24); TP by biuret method [U. Miami] vs. TS (n = 21); and ALB by bromocresol green method [Antech] vs. ALB by protein electrophoresis [U. Miami] (n = 41) (n = 41; S2 Fig). ALB by bromocresol green method [Antech] was significantly greater than ALB by protein electrophoresis [U. Miami] by 0.17 g/dL on average (S3 Fig). TP by biuret method measured at Antech and U. Miami were on average 0.34 and 0.41 units higher than TS measured by refractometry in-house, respectively. These differences were statistically significant (S3 Fig), and could be clinically relevant especially for hypoproteinemic turtles. TS are frequently performed in-house to estimate TP but are affected by increased concentrations of osmotically active analytes, such as Glc, Na, and BUN. The extremely low TS in DTs are primarily because the DTs had low TP but may also be caused in part by the very low Glc and BUN concentrations or inaccuracies of reading the refractometer at such low TP concentrations. The significantly greater ALB by bromocresol green method compared to ALB by protein electrophoresis in DTs is consistent with a recent report in diseased Hermann’s tortoises [35]. This is likely due to assay interference of substrates other than ALB, such as fibrinogen or globulins, in the bromocresol green method or inaccuracies at very low ALB concentrations [34, 35]. Similar to other reports, we emphasize the importance to consider these limitations of the bromocresol green method and to utilize protein electrophoresis if accurate ALB concentrations are desired in sea turtles.

The BUN concentrations demonstrated an increase through rehabilitation (D was greater than A-survived) to a degree that was significantly higher compared to HTs (S3 Table). Low initial BUN likely reflects chronic starvation and possibly hepatic insufficiency, while production of BUN increases through rehabilitation after acceptance of food and/or recovery of liver function after hemodynamic improvements from treatment and rehydration [6]. Higher BUN in rehabilitated DTs compared with HTs may reflect consumption of a higher protein and higher quantity diet by turtles in captivity compared with free ranging HTs. BUN concentrations higher than healthy free-ranging turtles have been seen in captive rehabilitated stranded loggerhead sea turtles [26] and convalescent cold-stunned Kemp’s ridley sea turtles [6].

The Ca: P ratio was lower in the DTs that died and significantly increased at time point B compared to HTs (S3 Table). P appeared to drive these differences more than Ca, as Ca was only mildly low at time of admission and B. P (Fig 4D) was highest in DT dead and A-died. It was observed at concentrations similar to HTs in A-survived, C and D, but a significant decrease was seen at B. In fact, P decreased without a concurrent Ca decrease, resulting in marked hypophosphatemia. This observation may be explained by increased urinary excretion after hemodynamic adjustments from rehydration/fluid therapy, and/or prolonged intestinal malabsorption or anorexia at time point B. Ca:P is used for diagnosis of renal disease in terrestrial reptiles [36], but caution is warranted in using this ratio in marine turtles. Ca and P homeostasis is often reversed and can be significantly influenced by dietary availability and bone growth in sea turtles [6]. Various factors need to be considered when interpreting this ratio in DTs. Hypoalbuminemia likely contributes to reduced protein-bound Ca fractions in plasma of DTs that died. It is ideal to use ionized Ca measurements for the assessment of the biologically available Ca concentrations [37], which were not performed in this study. Nutritional deficiencies or differences (wild vs. captive diet) may also contribute to hypocalcemia in DTs. High P concentrations at the time of stranding could be associated with reduced renal blood flow and decreased renal P excretion given the evidence of dehydration in DTs (visible sunken eyes, hyperuricemia). Muscle injury or wasting may also contribute to higher plasma P.

K was significantly higher in dead DTs and lower in all other DTs than HTs with some improvement between time points A and D (S3 Table). High K concentrations in dead DTs were possibly affected by post-mortem leakage of K from tissues in prolonged contact with plasma or from difficult blood withdrawal (this information was not recorded). Additional considerations include presence of metabolic acidosis, muscle injury, and/or decreased renal excretion of K. Possible explanations for the significantly lower K concentrations in other DTs include hyporexia or anorexia, and/or loss of K through kidneys and/or gastrointestinal tract.

Na and Cl were higher in dead DTs and lower at time point B compared to HTs (S3 Table). Likewise, UA was higher in dead DTs and A-died than HTs. This indicates that dehydration and/or renal insufficiency was present in the two most severe categories of DTs.

Elevated concentrations of certain enzymes in the plasma can indicate tissue damage. Plasma AST was higher in A-died and the B time point compared to HTs, and it was higher at admission than recovery (S3 Table). CK was significantly higher in A-died than recovered DTs or HTs. Both enzymes are widely distributed in tissues of loggerhead sea turtles, with highest concentrations of CK in heart, skeletal muscle, gastrointestinal tract and central nervous system, and highest concentrations of AST in heart, liver and kidney [38]. Concurrent elevations of both enzymes may indicate muscle injury/wasting or marked gastrointestinal disease [38]. Both of these organ systems are affected in debilitated turtles.

The low detection frequency of bile acids and lack of significant changes among DT categories (S3 Table) raises concern that the radioimmunoassay does not detect reptilian bile acids or alcohols and that a species-specific assay is needed. This is consistent with a recent study using the same assay in loggerhead turtles [39], and highlights the need for a reptile-specific test method.

During biliverdin measurements, the limit of detection (LOD) changed between the time when the DT samples (LOD = 0.6 mg/dL) and the HT samples (LOD = 1.25 mg/dL) were analyzed. This resulted in no detection of biliverdin in the HTs and challenges to interpret changes through rehabilitation. A-survived DTs had the highest biliverdin concentrations, and these were significantly higher than HTs (S3 Table). Of the three turtles admitted with green plasma, biliverdin measurements were available for two samples and these had the two highest concentrations, 4.7 and 9.3 mg/dl, indicating an association of biliverdin concentration and plasma discoloration. Reptiles lack biliverdin reductase and excrete biliverdin rather than bilirubin as in mammals, and thus this analyte has potential as a diagnostic indicator of compromised liver function. The assay, though, requires further optimization and validation.

### Immune functions

Measuring immune functions is relatively new to the field of sea turtle biology, and few studies have performed hematology along with multiple immune functions that span both the innate and acquired immunity [21, 40]. Data for lymphocyte proliferation (LP), a measure of acquired immunity, as well as respiratory burst and lysozyme activity, both measures of innate immunity, in the different turtle categories are shown in S4 Table. Respiratory burst activity is an indicator of innate immunity, infection, and inflammation. When assessed using NBT as described herein, it determines the production of superoxide formation from neutrophils, the cellular equivalents of reptilian heterophils, and monocytes [41]. Superoxide formation and release is critical in host defense to bacterial and parasitic infections and is increased in animals following antimicrobial challenges [42]. Plasma lysozyme activity is a marker of pro-inflammatory/inflammatory responses and a measure of innate immunity [43, 44]. Lysozyme is produced by fish and mammalian neutrophils in response to bacteria [43, 44]. Reptilian lysozyme can lyse and kill both gram-positive and negative bacteria [45]. Proliferation of T- and B-cells as measured by mitogen stimulation determines the ability of the adaptive immune system to mount a response as proliferation of these cells is a critical step in the process.

**Fig 5:**
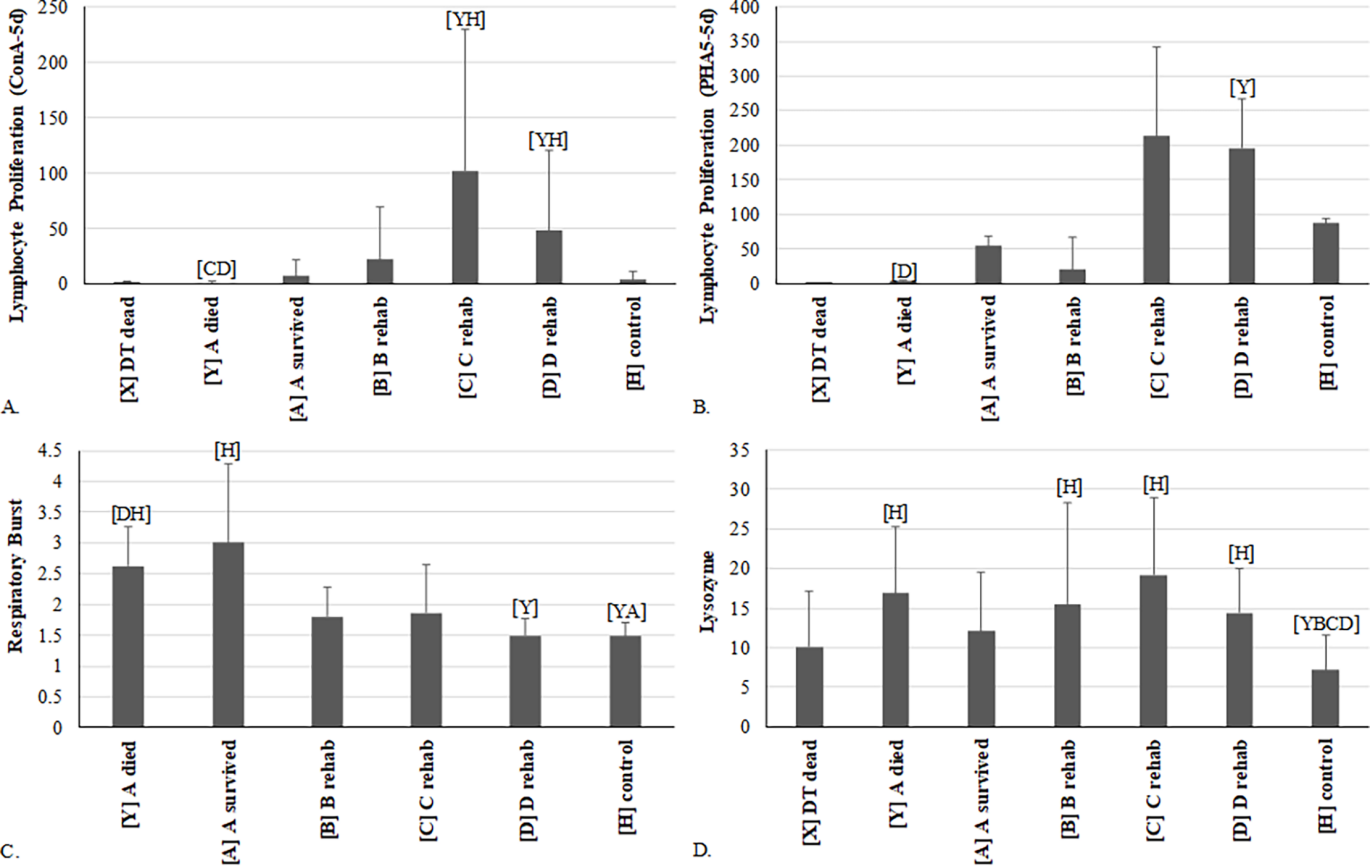
Comparison of immune function variables measured in debilitated loggerhead sea turtles (DTs) before and during rehabilitation and compared to healthy loggerhead turtles (means and one standard deviation). Each turtle group on the x-axis was significantly different (p<0.05 or p<0.00883 for repeated measures) from groups represented by letters above data. (A) lymphocyte proliferation with ConA; (B) lymphocyte proliferation with PHA; (C) respiratory burst with Ca ionophore; and (D) lysozyme activity. Lines represent an individual turtle through rehabilitation. For samples sizes in each diagram, please refer to S4 Table.

Both LPS- and PDB-induced LP were significantly suppressed in one or more of the DT categories at time of admission compared to HTs, while concurrently elevated respiratory burst and plasma lysozyme activity were also observed (S4 Table; Fig 5). Specifically, respiratory burst (super oxide production) was significantly elevated at admission but rapidly declined, such that no difference from HTs was observed by time point B (Fig 5C, S4 Table). Plasma lysozyme activity was significantly increased in DT compared to HTs throughout rehabilitation (Fig 5D, S4 Table) supporting the suggestion of ongoing inflammation, as observed by the described leukogram changes (i.e., left-shifting). Elevated innate immunity has also been demonstrated in dehydrated Gila monsters (*Heloderma suspectum*) and those undergoing fat store loss [46]. It was suggested that the reptiles were actively elevating physiologically cheaper innate immune response strategies during stressful periods. It is possible DTs are using similar immune response mechanisms.

Through rehabilitation, LPS- and PDB-induced LP did not significantly change between A and D sample time points, but was significantly decreased in A-died samples compared to HTs (S4 Table). This suggests suppression of humoral immunity to both T-dependent and T-independent antigens in these turtles at stranding. Increased innate immunity functions could be an attempt to compensate for this deficit and/or be linked to inflammation. Interestingly though, LP stimulated with ConA (T-lymphocyte proliferation) for 5 days exhibited an increase during rehabilitation to levels that were an order of magnitude higher than HTs at time points C and D (Fig 5A). The occurrence of compensation between branches of the immune system and the ability of the system to rebound after suppression from drugs, stress hormones, environmental stressors and malnutrition (fasting, starving, decreased protein intake) is well known [47–52]. Once the stressor is removed, the system will overcompensate to levels much higher than normal before returning to normal levels. Therefore, the observed increase in ConA-induced LP is most likely due to the rebound effect following the stressors associated with a prolonged disease state, malnutrition, and inflammation.

PHA-induced LP showed a similar pattern to ConA-induced LP through rehabilitation, but was not statistically different from HTs due to the generally higher standard deviation observed with PHA (Fig 5B, S4 Table). Additionally, plasma lysozyme activity and numbers of monocytes all exhibited increases from A-survived to C followed by a slight drop at D; thereby, mirroring ConA- and PHA- induced proliferation patterns. The leukogram and immune function results complement each other. At stranding, the innate immune functions (respiratory burst and lysozyme) of DTs suggest activation, which is consistent with changes in indicators of inflammation, including increased heterophil toxicity and left-shifting (S2 Table), all of which stabilized and/or improved consequently during rehabilitation. The suppression of the adaptive immune system (decreased LPS- and PDB-induced LP at admission) matches the lower total lymphocytes (S2 Table) and gamma-globulins (S3 Table) seen at stranding in the DTs.

Overall, the data suggest that at stranding the innate immune system of DTs is activated (increased respiratory burst and lysozyme at admission) likely due to an immediate need to fight increased presence of parasites and bacteria and/or to compensate for suppressed humoral immunity. B-cell function, as indicated by LPS- and PDB-induced LP, was reduced in DTs that died compared to HTs, but no significant difference was observed between A-survived and HTs. These comparisons indicate that the turtles with the worst prognosis have the most severe B-cell suppression. Lymphoid depletion and reduced antibody production has been associated with starvation in chicks [53]. A similar mechanism can be presumed in chronically debilitated turtles. Suppression of any part of the immune system can increase susceptibility to infections, specifically to parasites when lymphocyte defects are present [54]. However, T-cell functions seemed to rebound following admission. These findings provide new information on the immune responses of sea turtles and more generally in reptiles, all of which are understudied [45]. In particular the elevated and long-lasting increases in plasma lysozyme activity in DTs supports the idea that innate immunity antimicrobial proteins and associated mechanisms are potent and important defense mechanisms for reptiles [25, 45].

### Prognostic indicators

**Fig 6:**
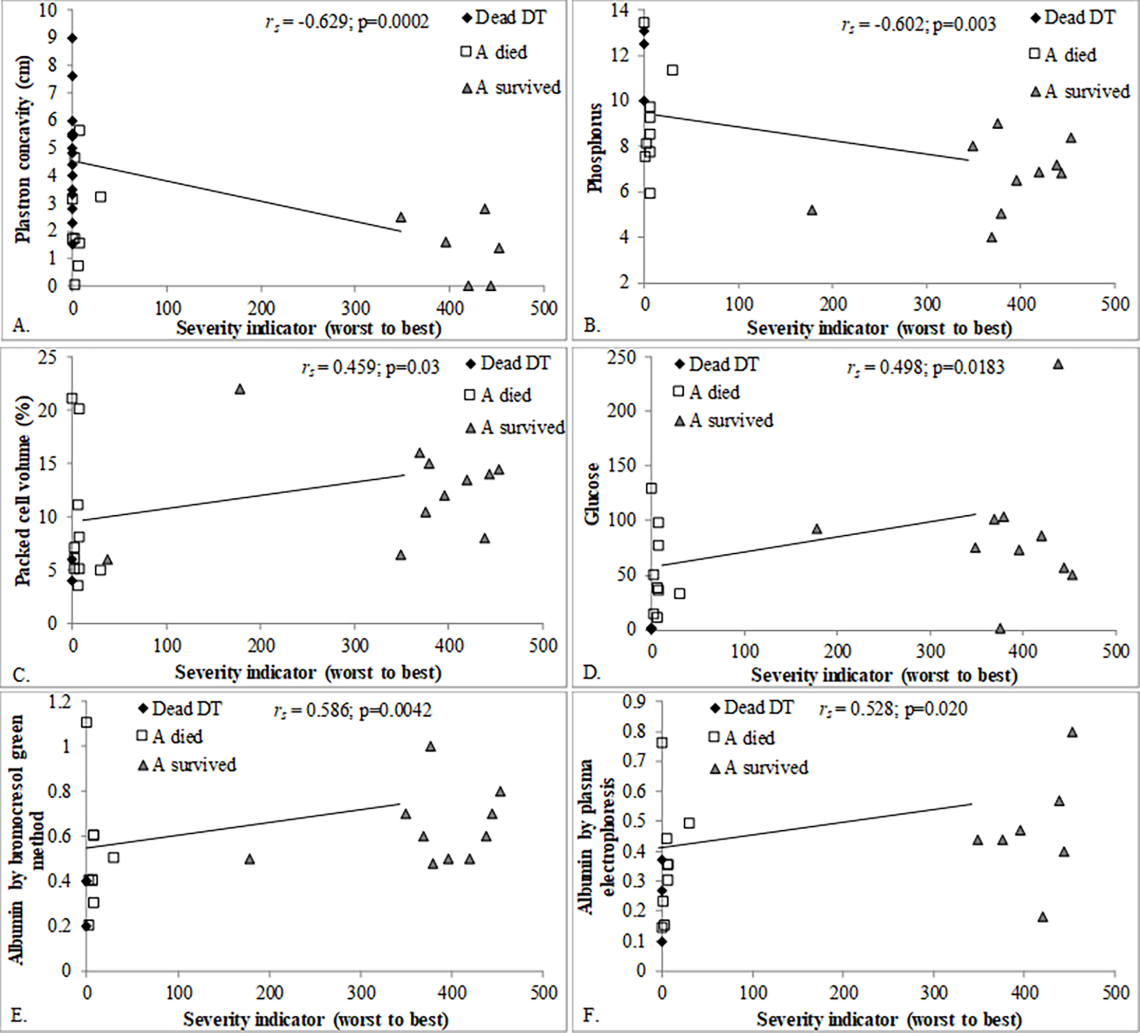
Health variables that significantly correlated with the severity indicator in debilitated loggerhead sea turtles (DTs). Spearman rank correlation coefficients and p-values are shown. (A) plastron concavity; (B) packed cell volume (PCV); (C) glucose; (D) phosphorus; (E) albumin by bromocresol green method; (F) albumin by plasma electrophoresis; (G) B-lymphocyte proliferation.

The severity indicator calculated for DT dead, A-died, and A-survived provided a continuous variable to examine correlations with other health indicators. Those that are significantly correlated (S5 Table) with the severity indicator are considered to be the most prognostic. Fig 6 visually shows the relationship for selected variables. Note that the most severe cases had the lowest severity indicator score (Fig 1). Analytes with a significant correlation to worsening severity included greater plastron concavity (Fig 6A), P (Fig 6B), UA, and K, and reduced PCV (Fig 6C), total WBC, Glc (Fig 6D), TS, TP by Antech, ALB by bromocresol green method (Antech, Fig 6E) and by protein electrophoresis (U. Miami, Fig 6F), lymphocyte proliferation at 4 days (PDB 25 and 50) and at 5 days (PDB 25), alpha 1-globulins, beta-globulins, Ca:P, and biliverdin. Based on statistical analyses and clinical judgement, plastron concavity ≤ 2 cm, PCV ≥10%, P ≤9 mg/dL, ALB ≥0.4 g/dL, UA ≤2.4 g/dL, Glc ≥55 mg/dL, and TS ≥2 g/dL are the most predictive of a DT surviving rehabilitation. Unfortunately, measuring plastron concavity on live DTs is not recommended because of the high risk of cardiac tears from plastron bones that are more freely moveable from reduced connective tissue in DT.

### Conclusions and clinical relevance

This study defined characteristic morphometrical and clinicopathological abnormalities of DTs at admission and through rehabilitation and identified clinically useful prognostic indicators. This new information is unique in that it reports a comprehensive database focused solely on DTs and sets itself apart from previous studies that have examined groups of loggerhead turtles that stranded from various causes [8, 11, 26].

The clinical diagnosis of chronically debilitated loggerhead turtles is made simply by visual assessment of emaciation, lethargy, and heavy barnacle coverage on skin. At time of admission, severe metabolic derangements are observed and reflected in hematological and plasma chemistry profiles. Live patients have a guarded prognosis, with a documented mortality of 52% in this study, which highlights the challenges for treatment and rehabilitation. Plastron concavity and PCV are variables with substantial prognostic significance, but we encourage development of innovative methods for taking plastron measurements on live DTs while they are in normal upright position rather than turning them to dorsal recumbency and risking severe injuries.

Improvements in treatment strategies can hopefully be made based on our descriptions of the severe systemic pathophysiological effects in DTs. The animals require long-term care with the goals to reverse the emaciated condition and treat parasitic and bacterial infections. Treatment of the individual patient is based on the veterinarian’s discretion, and the current consensus on best treatment strategies among experts include the use of a combination of crystalline and colloidal fluid therapy, total parenteral nutrition, and antimicrobial therapy early on in the course of rehabilitation [10]. Blood transfusions are reserved for severe cases (PCV ≤5%). Gradual introduction to de-beaked and de-boned seafood should be instituted. Once the turtle is eating regularly gastrointestinal protectants and oral antibiotics (metronidazole) are often used. Deworming agents such as fenbendazole should be utilized but not until the anemia has significantly improved and a regenerative response is noted [10].

These recommendations for intense medical treatment from the literature and the findings of this study demonstrate the severe clinical condition of chronic debilitation, representing the end stage of starvation. Future studies are needed to further understand the initial cause(s), temporal and spatial trends, and the effects on the loggerhead sea turtle population in the Southeastern United States.

## Supporting information

S1 Fig Photos of a debilitated loggerhead sea turtle. Photographs of a dead debilitated loggerhead sea turtle (Caretta caretta) with (A) emaciation, sunken eyes, epibiota on head and skin, and (B) severely sunken plastron, poor body condition, and generalized epibiota growth on plastron and skin.
(TIF)

S2 Fig Scatterplots of different measures of plasma proteins in debilitated loggerhead sea turtles (DTs). Linear trendlines and equations are shown for each comparison along with the Spearman correlation coefficient and p-value.
(TIF)

S3 Fig Differences between different measures of plasma proteins in debilitated sea turtles (DTs). Mean and standard deviation of the difference between paired plasma samples from debilitated loggerhead turtles (DTs) for different measures of plasma proteins. PE = plasma electrophoresis; BG = bromocresol green. An asterisk indicates a significant difference between the two methods compared within each bar (Wilcoxon signed rank test, p<0.05).
(TIF)

S1 Table Morphometric data in debilitated sea turtles (DTs) stranded along the southeast U.S. compared to healthy control turtles. (DOC)

S2 Table Hematological data in debilitated sea turtles (DTs) stranded along the southeast U.S. compared to healthy control turtles. (DOC)

S3 Table Plasma chemistry data in debilitated sea turtles (DTs) stranded along the southeast U.S. compared to healthy control turtles. (DOC)

S4 Table Immune function measurements in debilitated sea turtles (DTs) stranded along the southeast U.S. compared to healthy control turtles. (DOC)

S5 Table Prognostic indicators determined by correlations with severity indicator. Spearman correlations between the severity indicator in debilitated loggerhead turtles (DTs) and hematology, plasma chemistry and immune function variables. Values for the severity indicator decrease with worsening severity of debilitation. P-values in bold with * are significant.
(DOC)

S1 File Protocol for assessing deibilitated loggerhead sea turtles. Written protocol, datasheets, flowcharts.
(PDF)
