# Supplementary figures and images for: Chronic debilitation in stranded loggerhead sea turtles (Caretta caretta) in the southeastern United States: Morphometrics and clinicopathological findings
Source: PLoS One. 2018 Jul 10;13(7):e0200355. doi: 10.1371/journal.pone.0200355 (PMC6039040; doi:10.1371/journal.pone.0200355)

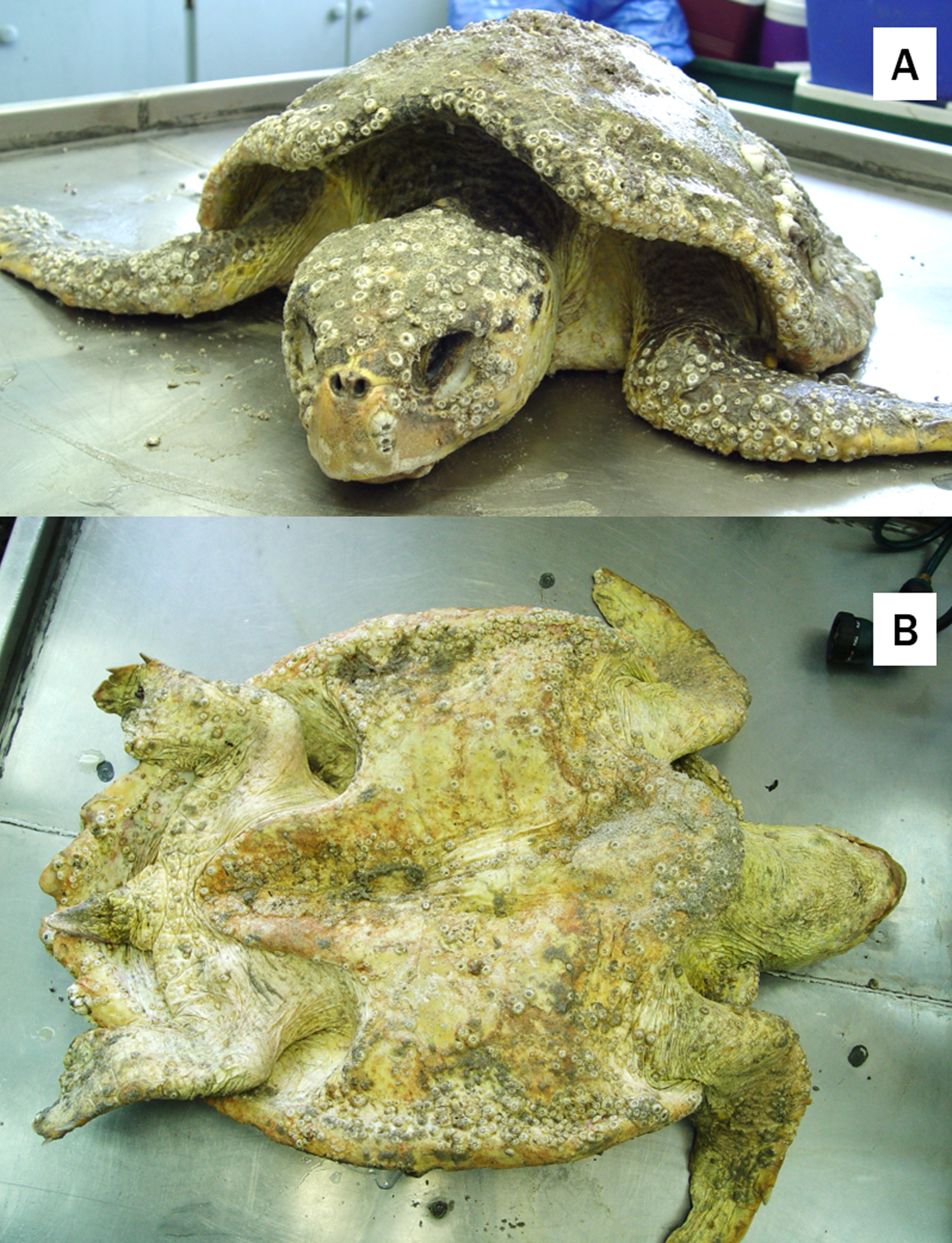

Supplement: S1 Fig — Photographs of a dead debilitated loggerhead sea turtle (Caretta caretta) with (A) emaciation, sunken eyes, epibiota on head and skin, and (B) severely sunken plastron, poor body condition, and generalized epibiota growth on plastron and skin. (TIF) [file pone.0200355.s001.tif]

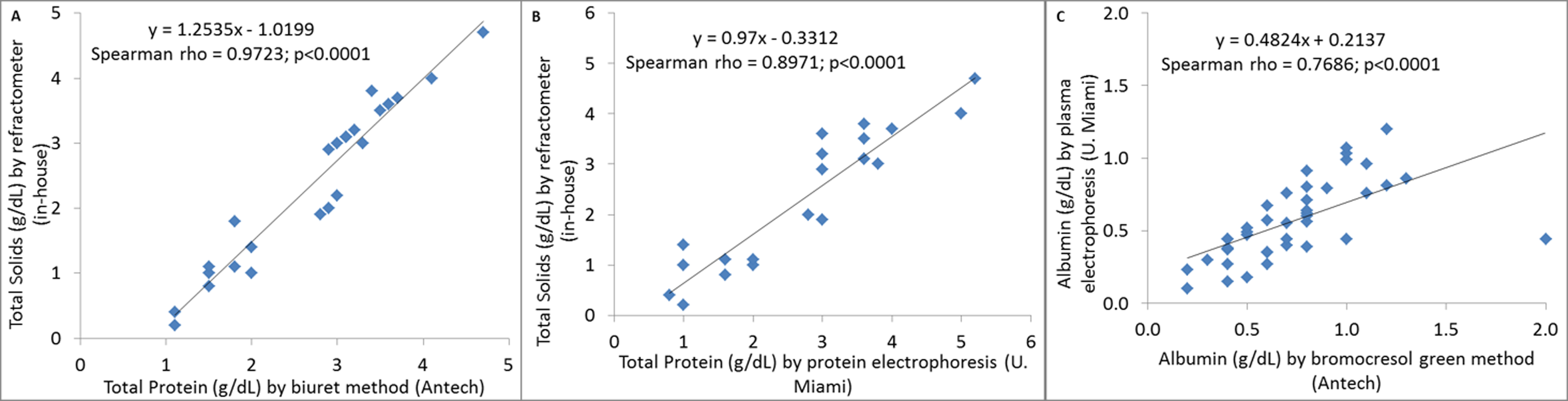

Supplement: S2 Fig — Linear trendlines and equations are shown for each comparison along with the Spearman correlation coefficient and p-value. (TIF) [file pone.0200355.s002.tif]

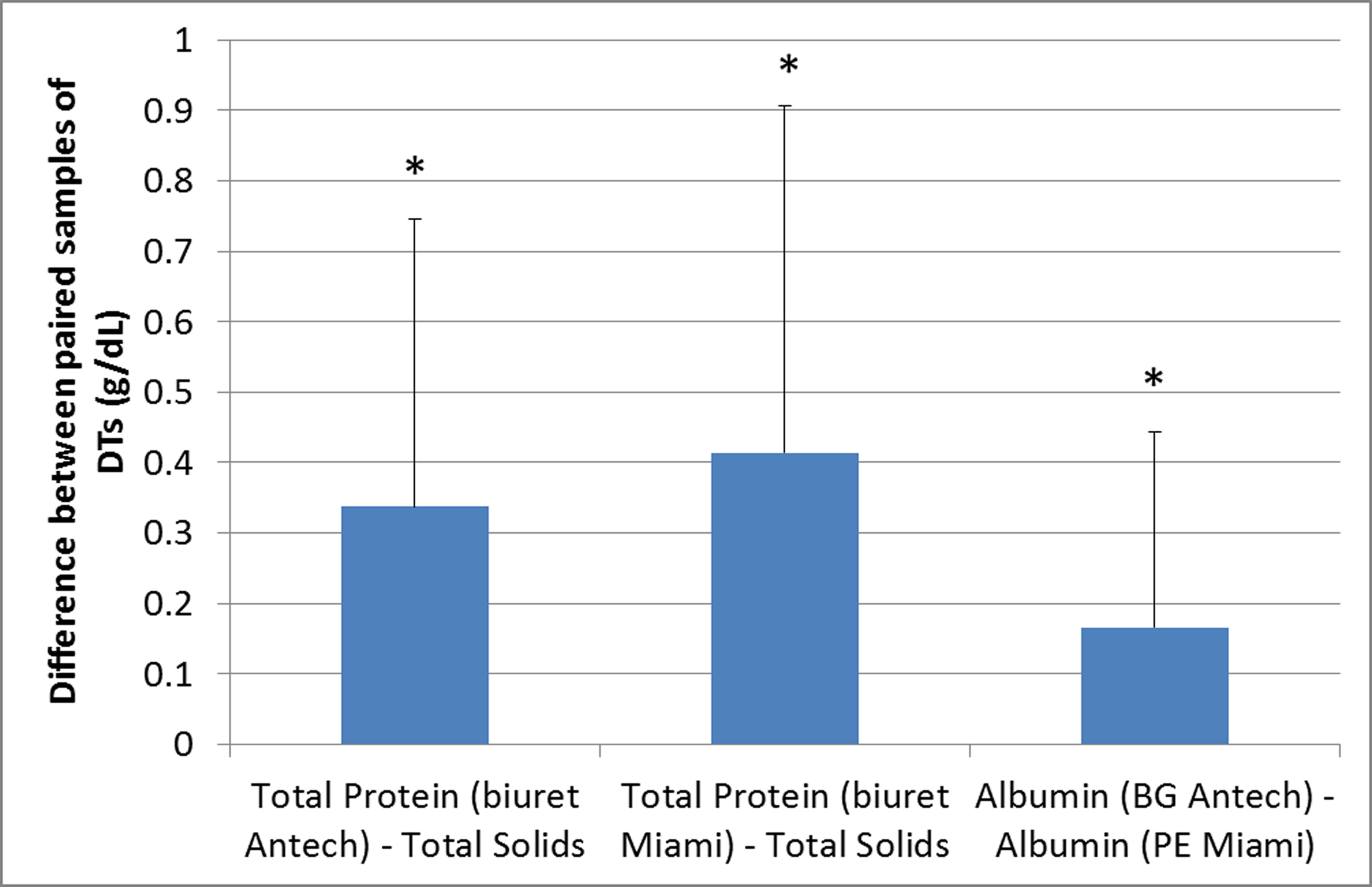

Supplement: S3 Fig — Mean and standard deviation of the difference between paired plasma samples from debilitated loggerhead turtles (DTs) for different measures of plasma proteins. PE = plasma electrophoresis; BG = bromocresol green. An asterisk indicates a significant difference between the two methods compared within each bar (Wilcoxon signed rank test, p<0.05). (TIF) [file pone.0200355.s003.tif]
