# Supplementary material for: Chronic debilitation in stranded loggerhead sea turtles (Caretta caretta) in the southeastern United States: Morphometrics and clinicopathological findings
Source: PLoS One. 2018 Jul 10;13(7):e0200355. doi: 10.1371/journal.pone.0200355 (PMC6039040; doi:10.1371/journal.pone.0200355)
Supplement: S3 Table — (DOC) [file pone.0200355.s006.doc]

Table S3. Plasma chemistry data in debilitated loggerhead sea turtles (*Caretta caretta*) stranded along the southeast U.S. compared to healthy control turtles.

^a^ Each category of turtle was assigned a letter found in brackets in the column headers. DT dead = debilitated turtles that were dead at time of stranding, sample collection at time of stranding; A-died = turtles that died shortly after stranding, sample collection at time of stranding; A-survived = turtles that survived and were released after successful rehabilitation, sample collection at time of stranding; B = sample collection from survivors approximately 1 week after beginning to eat; C = sample collection from survivors approximately 1 to 10 weeks after B sample; D = sample collection from survivors immediately prior to release. H = control turtles that represent apparently healthy turtles.

Statistically significant differences (p<0.05 or p<0.00883 for repeated measures) among DT categories are shown in brackets in the sample size rows. NSD = not significantly difference from any turtle category.
