# Supplementary material for: Chronic debilitation in stranded loggerhead sea turtles (Caretta caretta) in the southeastern United States: Morphometrics and clinicopathological findings
Source: PLoS One. 2018 Jul 10;13(7):e0200355. doi: 10.1371/journal.pone.0200355 (PMC6039040; doi:10.1371/journal.pone.0200355)
Supplement: S5 Table — Spearman correlations between the severity indicator in debilitated loggerhead turtles (DTs) and hematology, plasma chemistry and immune function variables. Values for the severity indicator decrease with worsening severity of debilitation. P-values in bold with * are significant. (DOC) [file pone.0200355.s008.doc]

Table S5. Spearman correlations between the severity indicator in debilitated loggerhead turtles and hematology, plasma chemistry and immune function variables. Values for the severity indicator decrease with worsening severity of debilitation. P-values in bold with * are significant.
