# Supplementary material for: Chronic debilitation in stranded loggerhead sea turtles (Caretta caretta) in the southeastern United States: Morphometrics and clinicopathological findings
Source: PLoS One. 2018 Jul 10;13(7):e0200355. doi: 10.1371/journal.pone.0200355 (PMC6039040; doi:10.1371/journal.pone.0200355)
Supplement: S1 File — Written protocol, datasheets, flowcharts. (PDF) [file pone.0200355.s009.pdf]

STANDARDIZED PROTOCOLS FOR EVALUATING DEBILITATED LOGGERHEAD SEA TURTLES (*Caretta caretta*)

I. BACKGROUND AND RATIONALE

In 2003, there was a perceived increased occurrence of emaciated and barnacle-laden loggerhead turtles (*Caretta caretta)* found stranded (both dead and moribund) along the southeastern US Atlantic coast. To investigate this situation further, the Wildlife Conservation Society's St. Catherines Island (SCI) Wildlife Survival Center (WSC) and the Georgia Department of Natural Resources organized a workshop on SCI in November of 2003. Fifteen people attended including: turtle biologists from Florida, Georgia, South Carolina, and North Carolina, veterinarians, toxicologists, immunologists, and representatives from the National Oceanic and Atmospheric Administration (NOAA).

The group determined that there was an increasing trend in strandings of debilitated sea turtles from 1992-2002 (approximately 11% annual increase). The number of debilitated turtles appeared to increase substantially in 2003 (NC 3%, SC 22%, GA 10%, and FL 22% of the total turtle strandings). The species composition of debilitated sea turtle strandings was primarily loggerheads, but a few green (*Chelonia mydas*), Kemp's ridley (*Lepidochelys kempii*) and possibly in Florida, a hawksbill (*Eretmochelys imbricata*) turtles were affected. Temporally, the stranding of debilitated turtles occurred all year in Florida; however, strandings were found to be concentrated in the spring and summer (April through July) in the other states. Spatially, debilitated sea turtles were stranded across the southeastern US coastal region, however, there were areas of high stranding density in the southern part of North Carolina, the northern part of South Carolina (Georgetown and Horry Counties) and around Cape Canaveral in Florida (Brevard County). Many explanations for stranding patterns were discussed including ocean currents, winds, and cold-stunning events.

A debilitated turtle was defined as emaciated with small barnacles covering the skin. The flippers may also have lesions or may be necrotic. While heavy epibiota can be a normal finding on the carapace and plastron of healthy loggerhead sea turtles, the skin is generally free of these commensals. Health assessment and necropsy data from these cases indicated the turtles were being affected by a wide range of secondary bacterial and parasitic infections with the primary cause still to be determined. Seven debilitated turtles showed significantly higher blood levels of polychlorinated biphenyls (PCBs) and organochlorine pesticides compared to 47 apparently healthy turtles. In a separate study, mercury concentrations in blood and scutes were 2 to 3 times higher in dead stranded turtles compared to live, apparently healthy turtles although the sample size was small. It is still unclear at what levels these compounds become toxic to sea turtles. The high contaminant levels could be a secondary effect as debilitated turtles use up their fat reserves, causing organic contaminants to become concentrated in blood.

The group determined several areas that need to be addressed in 2004. First, a complete statistical analysis of debilitated sea turtle stranding trends (NMFS-Sea Turtle Stranding and Salvage Network Database) is needed to better define the extent of the problem. This analysis will assist in determining if there was a substantial and statistically significant increase of stranded debilitated turtles in 2003. Possibly the strandings correlate with overall increases in offshore populations. Ongoing studies at the St. Lucie Power Plant in Florida, indicate a significant increase in loggerhead sea turtle populations. The average annual captures of this species from 1992 to 2002 was 275 turtles, while in 2003, 538 turtles were captured. Stranding reporting protocols will be reviewed to ensure that debilitated turtle strandings can be accurately assessed. In the past, not all strandings were examined for signs of debilitation. Thus the percentage of debilitated turtles should be expressed as a proportion of turtles examined, not total strandings. A second goal of this project is to standardize the health assessment performed on all of these turtles and store the data in a central location. And, thirdly, this study will further address contaminant and health data throughout the recovery of these debilitated turtles.

II. INTRODUCTION TO PROTOCOLS

In order to provide consistent, standardized documentation on stranded debilitated turtles, we have developed the following protocols which include: visual assessment, physical examination, morphometrics, clinical pathology, contaminant analysis, immune function tests, gross necropsy and histopathology. The accumulation and comparison of standardized health parameters will be critical in evaluating debilitated loggerhead sea turtles (*Caretta caretta)* and the cause(s) of their stranding.

Data may not be immediately available from banked samples or samples for contaminant analysis, because funding has not been allocated to this project and some of these analyses are extremely labor-intensive and costly (~$1000/sample). The samples will be stored properly by Jennifer (Keller) Lynch at NIST in expectation of future funding.

Introduction to Kit numbers and Datasheets:

Kits are compiled at MUSC and NIST with pre-cleaned supplies and supply from specific lot numbers. Kits for **live** debilitated turtles will be labeled as follows. For example, **DT-SC-R-1A** will be a debilitated turtle (DT) stranded in South Carolina (SC) that is alive (going to R for rehabilitation) and is the first live turtle sampled for this project (1). The A denotes the initial sampling event for that turtle. Kits DT-SC-R-1**B**, DT-SC-R-1**C**, and DT-SC-R-1**D** will be provided to the rehabilitation center for follow-up sampling.

Kits for sampling turtles during **necropsy** will be labeled similarly. For example, **DT-SC-N-1** will be a debilitated turtle (DT) from South Carolina (SC) that is sampled during necropsy (N) and is the first dead turtle (1) sampled for this project. Kits for control turtles will be labeled as **CTN-SC-1**.

Please follow this protocol. Once you are familiar with the details, you may find that the flow charts are helpful. Please fill out datasheets completely which are included in each kit.

Outline of protocols

Subsection	Page
Shipping and contact information	3
Case definition and documentation	4
Live debilitated turtle protocols	5-9
Supplied materials	5
Initial sampling procedures	6-9
Blood protocol	6-7
Blood collection	6
Blood contingency protocol	7
Blood handling	7
Blood shipping	7
Scute scraping	8
Fecal sampling	8
Epibiota sampling	8
Biopsy lesions/masses	8
Skin biopsy for genetics	9
Follow-up sampling procedures	9
Recovering sampling	9
Recovered sampling	9
Necropsy protocol for debilitated and control turtles	10-32
Necropsy sites	10
Toxicology sampling procedures	11-13
Gross necropsy protocol	13-14
Parasitology sampling	14-15
Epibiota sampling	15
Genetic sampling	15
Necropsy Report	16-33

III. SHIPPING AND CONTACT INFORMATION FOR BIOMATERIALS COLLECTION
[Contact information was provided in protocol, but has been removed for publication]

Blood samples must be shipped out on the same day as collected and arrive within 24-36 hours after collection. Please contact Margie to make arrangements.
This is **especially important** for shipping samples out on Fridays.

Immunology:
Margie Peden-Adams, PhD
Marine Biomedicine and Environmental Science Center (MBES)

Toxicology-Mercury (RD) and organochlorines (JK):
Jennifer (Keller) Lynch or Rusty Day
NIST-Charleston Laboratory
Hollings Marine Laboratory

Parasitolology:
Dr. Ellis Greiner, PhD
Dept of Pathobiology
College of Veterinary Medicine
University of Florida

Veterinarians:
Terry M. Norton, DVM, Diplomate ACZM
Wildlife Conservation Society
St. Catherines Island Wildlife Survival Center

Al Segars, DVM
South Carolina Department of Natural Resources
Marine Division

Craig Harms, DVM, PhD, Diplomate ACZM
North Carolina State University
Center for Marine Science and Technology

IV. Case definition for debilitated loggerhead sea turtles stranded live and dead.

Current terminology includes "barnacle bill," "living dead," and "severely debilitated." These terms include turtles that are emaciated, barnacle encrusted (carapace, plastron and skin), lethargic/non-responsive, and/or have skin/shell/underlying muscle necrosis and sloughing. Nonspecific terminology may encompass multiple syndromes, or represent a common end stage of multiple initiators, making comparisons between regions and years problematic. A broad case definition must be used initially, but linked with collection of data that can refine case definition in future investigations.

Minimum criteria:

- Grossly apparent emaciation--concave junction between neck and nuchal carapace with neck extended
- Concave plastron
- Sunken eyes
- May include severe edema (fluid accumulation under the skin) or ascites/anasarca (fluid accumulation in the coelomic cavity) masking signs of emaciation.

Accessory criteria:

- Extensive barnacle and other epibiota coverage and distribution
- Necropsy findings supportive of failure to feed (empty gastrointestinal tract, full gall bladder, fat depletion, serous atrophy of fat)
- Clinical pathology findings supportive of failure to feed may include some or all of the following: hypoproteinemia, hypoalbuminemia, low blood urea nitrogen (BUN), low blood glucose, and/or anemia.

Photos and Morphometrics:

- Because degree of emaciation and epibiota coverage are subjective judgments that could vary between observers, means to make objective comparisons must be pursued. Standardized digital photographs should be taken and collected centrally to assess epibiota load, degree of emaciation, and degree of skin sloughing if present. Use the provided **index card** to identify the turtle and include the following views:
  - **perpendicular views of:**
    - **entire carapace (dorsal-ventral)**
    - **entire plastron (ventral-dorsal)**
    - **head/foreflippers/cranial carapace (dorsal-ventral)**
  - **45 degree angle view of:**
    - **midsagittal craniocaudal view of head, neck and cranial carapace**
  - **lateral perpendicular view of side of head (right side for standardization)**
- **Send digital images to Dr. Terry Norton** at the email or mail address provided. Please use regular sized CD when sending images.
- Standardized morphometrics (SCL, CCL, SCW, CCW, depth/height, weight) should be taken to define the degree of emaciation objectively. Body weight and depth should be obtained whenever possible, as they are essential to any calculation of condition index. In addition to these measurements, take a plastron concavity measurement [***no longer recommended because of possible cardiac puncture***]. With turtle on back, measure distance from straight edge resting on widest point of ventral carapace to midpoint of sunken plastron (see diagram below). **Measurements to be taken can found on the datasheet provided.**

Plastron Concavity Measurement:

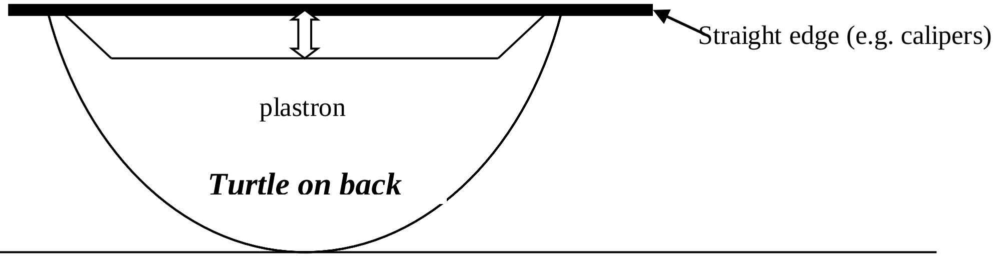

V. DEBILITATED LOGGERHEAD: <u>LIVE</u> ANIMAL PROTOCOL (Protocols 1&2 on flow charts)

The following diagnostic tools are recommended in all living loggerhead sea turtles encountered that fit the case definition of a debilitated turtle (page 4). If rehabilitation is the goal, then the clinician or rehabilitator responsible for the initial care of the animal will need to decide which samples can be safely taken without compromising the turtle's overall condition. **If the turtle can withstand 39 mL of blood collection, follow this protocol. If not, then please follow the "Blood Contingency Protocol (CP)".** If the turtle is to be euthanized, then please follow the blood sampling protocol described in this live animal protocol prior to administering euthanasia, and then continue with the necropsy protocol.

We would like to receive data on the following parameters, some of which will be performed "in house" or at your preferred laboratory and others will be sent to laboratories specified in the protocols: complete blood counts, plasma chemistry profiles, bacterial blood culture, plasma banking, genetics, immune assays, contaminant analysis, fecal parasite analysis, and biopsy and histopathology of suspicious lesions or masses. In addition, perform a complete physical examination and morphometric measurements, record current/relevant environmental conditions and trends in prey availability/ lack thereof in your region using the data collection form provided. Contaminants to be measured in certain samples are listed in Table 1 on a subsequent page.

<u>Supplied Materials:</u>
A sampling kit will be provided containing the following for each turtle and for each time point. The kit contents will include:

1. Alcohol swabs (2)
2. Vacutainer 21 gauge double-ended needles (3)
3. Vacutainer blood collection tubes (7 pre-labeled and numbered by collection order)
4. BBL Septi-chek blood culture tube (1) – some kits will not have this tube, if not, please **request blood culture tubes directly from Antech so that you have the tubes prior to any debilitated turtle stranding.**
5. Slides for making blood smears (4)
6. Slide holders (1 for Antech, 1 for Al Segars)
7. Plastic pipettes (3)
8. Corning cryovials for plasma from tube #5 ("BV", "BAPE", "BANK", "TEST") and tube #6 (plasma= "CHEM"; whole blood= "heparinized whole blood")
9. Vial containing lysis buffer for blood from tube #7 (1)
10. Bubble wrap
11. Disposable stainless steel 6 mm biopsy punch (2)
12. Pre-labeled polyethylene sample bag for scute scraping
13. Pre-labeled ziploc bags for fecal sampling (3)
14. Pre-labeled vials with formalin for epibiota samples (3)
15. Data Sheet
16. Pre-labeled index cards to use in photos for turtle ID
17. Shipping containers with return FedEx labels to Margie Peden-Adams (1), Antech (2), and Ellis Greiner (1)

A kit to be used between animals will also be provided and will contain:

1. Sharpie markers
2. Plastic scrubbing pad and plastic scraper (clean with 10% bleach or another appropriate disinfectant between each turtle)
3. Cleanroom wipers
4. Squirt bottle with high purity isopropanol
5. Squirt bottle with high purity water
6. Vials with formalin for lesion/mass biopsy
7. Shipping container with return FedEx label to Al Segars and Terry Norton for end of season samples
8. Antech forms with "Test Express" and proper tests selected
9. Gel packs (5 per turtle) – ***FREEZE THESE UPON RECEIVING KITS***

<u>Initial Sampling (Kit A):</u> Please follow the protocols described below for blood collection, scute scrapings, fecal sampling, biopsies, and epibiota sampling. Also, please fill out a datasheet. **Blood sampling from Kit A *<u>should also</u>* be performed on debilitated turtles prior to euthanasia.**

<u>Blood:</u> Blood should be drawn as soon as practical and prior to any therapy. Blood should be drawn through a **<u>double-ended needle</u>** directly into the blood tubes ***in the order in which they are labeled***. The order is important to minimize stress-related effects on immune function and to avoid trace element contamination.

Performing a complete blood count (CBC) and a plasma chemistry panel at this time is recommended. Packed cell volume, total protein, blood glucose, and any other abnormal parameters found on the initial sample should be performed once weekly. For consistency, we request that you use the following protocol and submit your samples to Antech Diagnostic Laboratory (AE160 Comp. Reptilian Profile and M060 Blood Culture). You have been sent submission forms and the charges will be billed to the project account. If you use your own institutional forms, the charges will be billed to your institution. Antech samples <u>must</u> be shipped by FedEx, and <u>not</u> by your local courier. Antech provides prepaid shipping containers and labels. If you use an alternative lab (not the preferred method), please note on the datasheet the lab being utilized and methodology used. We request that your blood analysis include: CBC, total protein, albumin, globulin, uric acid, BUN, Glucose, CPK, AST, Calcium, and Phosphorus.

<u>Blood Collection:</u>

1. Disinfect the neck first with betadine and then alcohol, beginning at venipuncture site and working outward. Allow the site to dry.
2. Collect blood using a double-ended needle in this order, gently inverting each tube five times after collection:

   #1: 7mL glass green-top tube (Na heparin in 2004 and lithium heparin in 2005): Full 7mL (immune function, metal analysis)
   #2: 5mL glass green-top tube (Na heparin): Full 5mL (metal analysis)
   #3: 5mL glass green-top tube (Na heparin): Full 5mL (organic contaminants)
   #4: 5mL glass green-top tube (Na heparin): Full 5mL or disregard if it will compromise the animal (organic contaminants)
   #5: 5mL glass green-top tube (Na heparin): Full 5 mL (bile acids, plasma protein electrophoresis, biliverdin, bank, metal analysis)
   #6: 5mL glass green-top tube (Na heparin): Full 5 ml (blood count, plasma chemistries, testosterone, metal analysis)
   #7: 3mL glass red-top tube (no additive): At least 0.5-1 mL unless a skin biopsy is taken (genetics)

Collect blood for bacterial culture prior to starting antibiotics. The blood culture tube (#8) should be sent to Antech. If not using Antech, check with your diagnostic lab for specific protocols (this will not be paid for by the project). Specimens for bacterial blood culture require collection into tubes provided containing growth medium (BBL, Septi-Chek TSB, Trypticase Soy Broth with SPS and CO_2). These tubes are available through Antech. Antech will process 1 tube for both aerobic and anaerobic organisms.

Blood culture collection procedure:

- A separate venipuncture site than above should be disinfected twice with an iodine preparation and then alcohol, beginning at venipuncture site and working outward. Allow the site to dry.
- Disinfect the rubber stopper of blood culture tube with alcohol.
- Collect 3 ml of blood into culture tube (vacuum in tube should fill to this level). Hold culture tube at room temperature (or up to 37 ºC) until transported/ shipped to lab.

Blood Contingency Protocol (CP) Collection:
If the turtle cannot withstand 39 mL of blood collection, then at the minimum collect the following tubes using a double-ended needle in this order and gently invert each tube 5 times after collection:

7mL Green-top tube #1: Full 7mL (immune function, OC analysis, metal analysis; LiHeparin 2005)
5mL Green-top tube #2: Full 5 mL – to replace tubes #2,5,&6 (bile acids, plasma protein electrophoresis, biliverdin, testosterone, bank, metal analysis, blood count, plasma chemistries)
Blood culture tube #8: After disinfecting new prep site.

Blood Handling:

1. Before blood clots, transfer whole blood from tube #7 (if collected) into a vial of lysis buffer via clean, unused pipette. Gently invert this vial several times.
2. Record approximate volumes collected in each tube on datasheet.
3. Store tube #8 at room temperature.
4. Keep all other tubes cool in a refrigerator or on wet ice until processed. If using wet ice, wrap tubes in bubble wrap or use another barrier (i.e. a styrofoam cup) to keep them from directly touching the ice in order to minimize hemolysis.
5. Gently invert tube #6 (or tube #2 in CP) five times. Make 4 blood slides: 2 for Antech and 2 for Al Segars (later shipped to Nicole Stacy). Allow slides to dry and store in slide holders and label holders with the date.
6. Transfer 0.3 mL whole blood from tube #6 (tube #2 in CP) via clean, unused pipette to cryovial labeled "heparinized whole blood". Recap tube #6 (tube #2 in CP). Label cryovial with date and refrigerate.
7. Centrifuge remaining blood in tube #6 and tube #5 (or only tube #2 in CP) for 5 minutes. Label the 5+ cryovials with the date.
8. Transfer 0.6 mL plasma from tube #6 (tube #2 in CP) via clean, unused pipette to cryovial labeled "CHEM." Transfer remaining plasma to cryovials "TEST" and "BANK." Ensure that all plasma is removed, recap tube #6 and refrigerate remaining red blood cells.
9. Transfer plasma of tube #5 (tube #2 in CP) via pipette to 3+ labeled cryovials:
   0.5 ml into "BAPE" (bile acids and protein electrophoresis)
   0.2 ml into "BV" (biliverdin)
   remainder into cryovials labeled "BANK" (banking) – fill equally
10. Ensure that all plasma is removed from the red cells in tube #5, recap tube #5, and refrigerate remaining red cells.

Storage prior to shipping:

11. Refrigerate: tube #1-4; remaining red cells in tube #5-6; blood lysis vial; cryovials labeled "heparinized whole blood," "CHEM," "BAPE," "BV," "TEST," and "BANK."
12. Room temperature: blood slides, blood culture tube #8.

Shipping (same day):

13. Contact **Margie Peden-Adams** on the same day of collection. Place plasma cryovials ("BAPE," "BV," "TEST," "BANK"); scute scrapings (see protocol below); and blood lysis vial or skin biopsy at the bottom of the shipping container. Place frozen gel packs directly on top of these samples. Wrap blood tubes #1-6 in bubble wrap so that tubes are not touching each other and place them on top of the gel packs. Place 2 of the blood slides "Al Segars" at the top of the container. Ship by FedEx overnight to Margie Peden-Adams.
14. Ship the cryovials labeled "heparinized whole blood" and "CHEM", 2 blood slides ("Antech") and the blood culture tube #8 (place this tube farthest away from the gel pack) by FedEx overnight to **Antech** with a frozen gel pack.

Scute scrapings: The scute scraping should be performed as soon after the blood collection as is practical. The scute scraping should be collected, stored, and shipped with the initial blood sample and will only be taken at the initial sampling phase (kit A only). The scute sample must be collected from the outermost edge of the eight most posterior marginal scutes of the carapace. Scutes free of fouling organisms/epibiota should be targeted for sampling.

1. Scrub 2 cm or more of carapace dorsal and ventral to the edge of these scutes vigorously with plastic scrubbing pad to remove sloughing keratin. If there are no areas free of epibiota, use the plastic scraper to clear the target area as thoroughly as possible prior to scrubbing.
2. Rinse the scrubbed area with high purity distilled water and isopropanol provided in the kit.
3. Remove remaining foreign matter and debris using clean room wipers, distilled water and isopropanol.
4. Hold a polyethylene sample bag under the prepared area.
5. Remove the lateral edge of the prepared marginal scutes by moving a disposable stainless steel biopsy tool parallel to the edge being sampled, allowing the shavings to fall directly into the polyethylene sample bag. Typically the posterior lateral corner of each scute will yield the thickest sample without penetrating the keratin and contaminating the sample with untargeted tissue. This should yield small shavings or splinters of keratin ~ 1 mm in thickness totaling 0.2-0.5 g. (Target approximately 4 inches or 10 cm total of 1 mm thick shavings).
6. Label the outside of the bag with the date.
7. Keep this sample cool in a refrigerator or on wet ice and ship with the blood samples on frozen gel packs.

***Fecal samples*:** A fecal sample for parasitology should be taken opportunistically prior to deworming (kit A) and at the end of rehabilitation prior to release (kit D).

1. Collect small amount feces (~one tablespoon) and place in a Ziploc bag. It is appropriate to collect feces from the water if there is still some consistency to it.
2. Double-bag sample and label both Ziploc bags with the date.
3. Place fecal sample in cooler on ice or in refrigerator (do not freeze!).
4. Ship sample with frozen gel packs (place the double-bagged sample into a 3rd Ziploc bag that contains the ice pack) via FedEx 2 day mail to Dr. Ellis Greiner within 7 days.

Epibiota samples:

1. Collect representative species and samples of epibiota from plastron, carapace, and skin.
2. Place in 3 separate vials (one for each location: plastron, carapace, skin) with 95% ethanol (preferred) or isopropyl alcohol and store at room temperature.
3. Ship to Terry Norton at the end of the season (November).

***Biopsy/histopath of suspicious lesion or mass*:**

1. Label formalin vial with identification number of turtle and specific location of mass/lesion. Please obtain photograph (preferably digital, labeled with animal ID, date, and stranding location) of lesion/ mass/biopsy.
2. Provide a brief description of lesion on the datasheet and other pertinent history of case should accompany sample to lab.
3. Representative tissue samples should be less than ¼ inch thick to ensure proper fixation. Proper fixation is important in preserving specimens for histopathologic evaluation. Ideally, tissue should be fixed in a volume of formalin solution 10 times the volume of the tissue for adequate fixation.

4. Send digital images and biopsy specimens to Dr. Terry Norton via regular mail (not paid for by project funds). These samples will be forwarded to Dr. Nancy Stedman for histopathological evaluation. Results will be available to clinician in a timely fashion.

***Skin sample for genetics*:**

1. If blood tube #7 was not collected for genetics, collect a tissue sample from trailing edge of flipper via a fresh disposable biopsy punch.
2. This can be done when the turtle is more stable.
3. Store tissue in 95% ethanol at room temperature and label vial with "DT genetics", turtle ID, date, and stranding location.
4. Ship samples to Al Segars at the end of the season, they will be forwarded to Joe Quattro at University of SC for analysis.

Follow-up sampling of live debilitated turtles in rehab (Kits B-D):

"Recovering Turtle" Samples (Kits B and C): Blood sampling should be repeated two times during the recovering phase of the animal. Ideally the chosen time points will capture the rate of maximum improvement, not after the animal has substantially improved.

B. The second samples (kit B) should be taken approximately one week after the turtle ***begins to eat*** food on its own.

C. The third samples (kit C) should be taken ***within 10 weeks of the second sample and after a small observable improvement*** in weight, body condition, behaviors, and clinical data parameters. Do not wait until the animal has fully recovered.

Use the kits labeled B and C with the turtle's original kit ID number. Follow the blood protocol described above to collect blood tubes #1-6. If the turtle cannot withstand 32 mL of blood collection, then follow the blood contingency protocol described above. Process and ship all samples as described above. No scute, fecal, blood culture, epibiota, or "TEST" samples are requested at these time points (supplies for those purposes are not included in kits B and C). Please fill out a datasheet at each sampling event and include in the shipment of samples. We realize that the second time point is vague and subjective, therefore recording weights, measurements, behavioral observations, and clinical data (i.e. PCV, TP, and other plasma chemistries) throughout the recovering phase will be very important. Please use and keep the "Major Milestones and Clinical Records" datasheet throughout treatment of the animal and include only in the final shipment of samples.

"Recovered" Sample: Just prior to release of the animal, repeat the blood, blood culture, and fecal sample collection. Use the kit labeled D with the turtle's original kit ID number to collect blood (tubes #1-6; 8) and fecal samples. Process and ship all samples as described above utilizing supplies in the kit, including "TEST". Please send copies of the "Major Milestones and Clinical Records" datasheet to Margie Peden-Adams, as well as any other available information, such as medical treatment and feeding schedules, and records of measurements, behavioral observations, and clinical data.

If the turtle dies during rehabilitation, follow the necropsy protocol – page 10.****

VI. DEBILITATED LOGGERHEAD: <u>NECROPSY PROTOCOL</u> (Protocols 3&4 on flow charts)

1) Primary Gross Necropsy Sites:

a) Turtles found in **North Carolina** and **northern South Carolina** (Georgetown and Horry counties) may be transported to:

- Dr. Craig Harms
  North Carolina State University
  Or
- Dr. Greg Lewbart or Shane Christian
  Department of Clinical Sciences
  North Carolina State University
  College of Veterinary Medicine

b) Turtles found in **southern South Carolina** (Charleston, Beaufort and Colleton) and **Georgia** will be transported to the Georgia Department of Natural Resources in Brunswick, GA or arrangements can be made through Dr. Terry Norton to drop off the turtle at St. Catherines Island Wildlife Survival Center dock in Midway, GA. Please contact either:

- Mark Dodd
  Georgia Department of Natural Resources
  Or
- Dr. Terry Norton
  St. Catherines Island Wildlife Survival Center

c) For turtles found in **Florida**, contact Terry Norton to discuss appropriate necropsy sites.

2) Toxicology Protocol for Collection of Tissues at Necropsy (Protocols 3&4 on flow charts)

<u>Strategy:</u> Contaminants to be measured in certain samples are listed in Table 1. Tissue samples will be collected from fresh dead (code 1) or euthanized debilitated turtles during necropsy (Protocol 3 on flow chart). If the debilitated turtle is to be euthanized, then blood samples should be taken using a live turtle Kit A prior to administering euthanasia, and additional blood collected during the necropsy will not be needed. Blood sampling from kit A will need to be processed and shipped out on the same day. If these blood samples were not collected, please collect blood during the necropsy as described below.

We are also in need of samples from **<u>control</u>** turtles: code 1 or euthanized **acute mortalities** (boat strike, entanglement) that appear to be otherwise "**healthy**" turtles (Protocol 4 on flow chart). If the control turtle will be euthanized, collect blood from the dorsocervical sinus using the supplies in the necropsy kit (not kit A). Blood does not need to be sent overnight. Follow the necropsy protocol and collect all tissues, including those for histopathology.

<u>Supplied Materials:</u>
A sampling kit will be provided containing the following for each dead stranded animal:

- Blood collection tubes (4 sodium heparin, glass, pre-labeled and numbered by collection order)
- Vacutainer double-ended needles, 21 guage (2)
- Stainless steel 6mm biopsy punch (disposable)
- 3 Pre-labeled polyethylene sample bags for scute, liver, and brain samples
- 2 pieces of pre-cleaned aluminum foil for fat and liver samples in separate Ziploc baggies
- 1 50 mL tube for additional liver (PFOS) sample
- 3 hexane-rinsed, foil wrapped scalpel handles and forceps
- 1 set of "No Teflon" handles and forceps for the additional liver (PFOS) sample
- Scalpel blades (4)
- Datasheet

A kit to be used between animals will include:
- Plastic scrubbing pad (clean with 10% bleach or another appropriate disinfectant between each turtle)
- Cleanroom wipers
- Squirt bottle with high purity isopropanol
- Squirt bottle with high purity water

<u>Sampling:</u> Blood, scute, fat, liver, and brain will be collected using the following protocols. Please collect these samples ***prior to removing organs or sampling for other purposes***. Samples should be collected ***in the order described*** to reduce contamination from other tissue types and external sources. Keep all samples cool in a refrigerator or on wet ice until they can be frozen. Freezing immediately is preferred. Please record all turtle ID information, all sampling problems, and storage locations on the provided datasheet.

<u>Scute scrapings:</u> The scute sample must be collected from the outermost edge of the eight most posterior marginal scutes of the carapace. Target scutes within this area that are free of fouling organisms.

1. Scrub 2 cm of carapace dorsal and ventral to the edge of these scutes vigorously with the plastic scrubbing pad to remove sloughing keratin. If there are no areas free of epibiota, use the plastic scraper to clear the target area as thoroughly as possible prior to scrubbing.
2. Rinse the scrubbed area with high purity distilled water and isopropanol.
3. Remove remaining foreign matter and debris using clean room wipers, distilled water and isopropanol.
4. Hold a polyethylene sample bag under the prepared area.
5. Remove the lateral edge of the prepared marginal scutes by moving a disposable stainless steel biopsy tool parallel to the edge being sampled, allowing the shavings to fall directly into the polyethylene sample bag. Typically the posterior lateral corner of each scute will yield the thickest sample without penetrating the keratin and contaminating the sample with untargeted tissue. This should yield small shavings or splinters of keratin ~ 1 mm in thickness totaling 0.2-0.5 g. (Target approximately 4 inches or 10 cm total of 1 mm thick shavings).

6. Label the outside of the pre-labeled bag with the necropsy date. Place the bagged sample inside a second outer bag and include a pre-labeled paper label inside the outer bag.
7. Freeze this sample at -20 ^{o}C or below.

Fat: Fat may not be available on these debilitated turtles. Occasionally, you may see black flaccid tissue in the inguinal regions where the fat is usually present. This is thought to be remnants of fatty tissue, please sample this if nothing else is available.

1) Open a pre-cleaned piece of foil completely, keeping the cleaned inside surface facing up.
2) Immediately after removing the plastron, use the provided scalpel blade, hexane-rinsed scalpel handle, and hexane-rinsed forceps to remove a 2 g to 5 g piece of fat from the **left inguinal region**. If little fat is available, take from other areas but note these locations on the datasheet.
3) Using the forceps, transfer the fat to the center of the pre-cleaned piece of aluminum foil.
4) Fold the foil in half over the tissue and make a closed pouch by folding over the 3 open edges several times onto themselves.
5) Place the sample inside a pre-labeled plastic Ziploc bag and label the outside with the necropsy date.
6) Freeze this sample at -20 ^{o}C or below.

*****Do not reuse this blade, handle or forceps to collect the liver sample.***

Liver: Three samples of liver will be collected for different purposes, therefore they must be collected and stored differently. *****Please don't puncture the gallbladder before taking these samples. And, do not touch the liver surface with plastic gloves or any object before taking these samples.***

1. Open a hexane-rinsed piece of foil completely, keeping the cleaned inside surface facing up.
2. Immediately after removing the plastron, use a fresh, provided scalpel blade, hexane-rinsed scalpel handle, and hexane-rinsed forceps to remove a one inch cube of liver from the **posterior marginal edge of the right lobe**.
3. Using the forceps, transfer the liver to the middle of the opened piece of aluminum foil (liver sample #1, foil).
4. Fold the foil in half over the sample and make a closed pouch by folding the 3 open edges several times onto themselves.
5. Place the foiled sample inside a pre-labeled Ziploc bag.
6. Using the same instruments, collect another one inch cube of liver from near the same location as the first sample.
7. Using the same forceps transfer the tissue to a pre-labeled polyethylene sample bag and seal the bag (liver sample #2, polyethylene bag).
8. Label the outside of both pre-labeled bags with the necropsy date.
9. Using a new scalpel blade with the handle and forceps labeled "No Teflon," collect a one inch cube of liver from the **posterior marginal edge of the left lobe**.
10. Place this sample into the 50 mL tube labeled "PFOS liver" (liver sample #3, 50 mL tube).
11. Freeze all 3 samples at -20 ^{o}C or below.

*****Do not reuse these blades, handles or forceps to collect the brain sample.***

Blood: If the turtle was euthanized, blood should have already been collected. If not, and the heart is still beating, then:

1. Place one end of the **double-ended needle** into the heart.
2. Collect tubes #1 and #2 (5mL green-top tubes for organic contaminants) followed by tubes #3 and #4 (5mL green-top tubes for metal analysis).
3. Try to get at least 5mL total for each of the two contaminant analyses.
4. Label tubes with necropsy date.
5. Keep tubes on ice, centrifuge them to separate plasma from red blood cells, and freeze them standing upright at -20 ^{o}C as soon as possible.

Brain:

1. Open the brain cavity carefully to avoid touching the tissue with your instruments.
2. With a fresh, provided scalpel blade, hexane-rinsed scalpel handle, and hexane-rinsed forceps, cut the brain in half laterally. Transfer one half directly to a polyethylene sample bag. The other half of the brain should be saved in formalin for histopathology.

3. Label the outside of the pre-labeled bag with the necropsy date. Place the bagged sample inside a second outer bag and include a pre-labeled paper label inside the outer bag
4. Freeze the sample at -20 °C or below.

Storage and shipping:
Store all toxicology samples frozen. Blood tubes cannot be stored at temperatures below -20 °C or they will break. Toxicology samples and datasheets collected at necropsy should be shipped ***on dry ice*** to Jennifer Keller at the end of the season (November).

Table 1. List of contaminants targeted for analysis in certain tissues.

Total Mercury
IN:
brain
blood
scute
liver

PFCs
PFOS
PFOA
IN:
liver
plasma

Other organohalogens				
PCB congeners			Organochlorine pesticides	PBDE congeners
PCB 1	PCB 114	PCB 177	alpha-HCH	PBDE 17
PCB 8	PCB 118	PCB 178	beta-HCH	PBDE 25
PCB 18	PCB 119	PCB 180	gamma-HCH	PBDE 28
PCB 28	PCB 121	PCB 183	HCB	PBDE 30
PCB 29	PCB 126	PCB 185	aldrin	PBDE 33
PCB 31	PCB 127	PCB 187	dieldrin	PBDE 47
PCB 44	PCB 128	PCB 188	endrin	PBDE 49
PCB 45	PCB 130	PCB 189	mirex	PBDE 66
PCB 49	PCB 132	PCB 191	*cis*-chlordane	PBDE 71
PCB 50	PCB 137	PCB 193	*trans*-chlordane	PBDE 75
PCB 52	PCB 138	PCB 194	*cis*-nonachlor	PBDE 85
PCB 56	PCB 146	PCB 195	*trans*-nonachlor	PBDE 99
PCB 63	PCB 149	PCB 196	heptachlor	PBDE 100
PCB 66	PCB 151	PCB 197	heptachlor epoxide	PBDE 116
PCB 70	PCB 153	PCB 199	oxychlordane	PBDE 119
PCB 74	PCB 154	PCB 200		PBDE 138
PCB 77	PCB 156	PCB 201	4,4'-DDT	PBDE 153
PCB 79	PCB 157	PCB 202	2,4'-DDT	PBDE 154
PCB 82	PCB 158	PCB 203	4,4'-DDE	PBDE 155
PCB 87	PCB 159	PCB 205	2,4'-DDE	PBDE 156
PCB 92	PCB 163	PCB 206	4,4'-DDD	PBDE 181
PCB 95	PCB 165	PCB 207	2,4'-DDD	PBDE 183
PCB 99	PCB 166	PCB 208	endosulfan I	PBDE 190
PCB 101	PCB 167	PCB 209	endosulfan II	PBDE 191
PCB 104	PCB 169		endosulfan sulfate	PBDE 203
PCB 105	PCB 170	***IN:***		PBDE 205
PCB 106	PCB 172	***fat***	**Other organochlorines**	PBDE 206
PCB 107	PCB 174	***liver***	octachlorostyrene	PBDE 209
PCB 110	PCB 175	***plasma***	pentachlorobenzene	
PCB 112	PCB 176			

3) Gross Necropsy Protocol

Please follow the necropsy form provided below that contains ***reminder boxes*** for collecting the toxicology and other samples; however, the original form can be downloaded from the following website:

http://www.vetmed.ufl.edu/sacs/wildlife/seaturtletechniques/necropsy

Use this form and follow it as closely as possible. The protocol is cumbersome, but results in a complete evaluation of all systems. The order of organ systems on the original form has been changed here to more closely match the order in which the necropsy is performed. Aerobic and anaerobic bacterial and fungal cultures should be performed when appropriate (not paid for but we would appreciate any results). Minimally, affected tissues should be frozen for future culture. A set of

representative tissues (thin sections) from all major organ systems listed on the necropsy datasheet should be collected and placed in 10% buffered formalin and a separate set frozen for future infectious disease cultures at -20˚C or below. A set of formalinized tissues, paraffin blocks, or representative slides should be sent to Dr. Terry Norton from each case. Please send the address where the final histopathology report should be sent. The frozen set of tissues should also be sent to Dr. Norton.

Dr. Terry Norton will submit the formalinized tissues to Dr. Nancy Stedman at Athens Diagnostic Laboratory, University of Georgia College of Veterinary Medicine. Dr. Stedman will evaluate all of the sets of tissues histologically. This will allow for more standardized results. Clinicians should feel free to submit another set of tissues to your pathologist of choice.

Supplied Materials:
Tissue cassettes labeled for each organ for tissues in formalin
Specimen cups to store tissue cassettes in formalin (3)
Plastic tubes labeled for each organ for frozen tissue
Plastic baggie to combine all frozen samples into (1)
4 microscope slides 2 slide holders for marrow impression smears
Syringe with a 20 gauge needle and 50 mL tube for bile collection
Overnight FedEx label for shipment of bile to Tom Sheridan
Formalin, because it cannot be shipped, and necropsy knives/chisels are not supplied

Special attention/instructions:

- When collecting gastrointestinal tract samples, take care to not touch the mucosa (inner lining) with anything (gloves, forceps, etc). Mucosa needs to be intact.
- Bone marrow is needed to further define the anemia that is observed in these turtles. To collect this, chisel between a marginal and lateral scute on the carapace. Remove a piece of trabecular (spongy looking, either red or yellow) bone within the lateral scute and place it in formalin. Record the color of the trabecular bone on the datasheet. Take another piece of this bone to make four marrow impression smears on four microscope slides by gently pressing the spongy material onto the slides. Don't be too rough with the slides or the cells will lyse. Allow slides to air dry and replace them in the cardboard slide holders. Place marrow smears inside multiple sealed baggies so that formalin does not come in contact with them. Send the formalinized sample and smears to Dr. Terry Norton with the other formalinized samples. Store another piece of bone in a plastic tube frozen for future infectious disease cultures and ship these along with the frozen set to Dr. Norton.
- For necropsies occurring in South Carolina only: Bile should be collected after the liver toxicology samples have been collected. Draw as much as possible into the provided plastic syringe and transfer bile into 50 mL tube. Refrigerate sample (do NOT freeze) and deliver same day with frozen gel packs to Tom Sheridan, Folly Road Animal Hospital,. This sample will be used to identify possible indicators that can further define the anemia that is observed in these turtles.

Storage and shipping:
Store all formalinized tissues in formalin, keep the marrow smears away from formalin, and keep the frozen set at -20 ^{o}C or below. Send all samples to Dr. Terry Norton at the end of the season. The formalinized tissues and smears can be shipped together as long as the smears are bagged in waterproof baggies. The frozen samples should be shipped ***on dry ice*** overnight.

4) Parasitology:

Supplied Materials:
50 mL tubes for parasite collection (3)
2-day FedEx label to ship samples to Ellis Griener (don't use this on a Friday)

Always keep the collected parasite specimens wet immersed in water, saline or fixative. Place worms from different organs into different labeled containers initially in physiological saline (0.85% NaCl). Each label

should indicate host species, accession or necropsy number, organ from which worm was removed, and collector's name. If there are many specimens, provide many specimens. Roundworm infection may be mixed and you are trying to provide both sexes of as many species as are present. Fluke infections also may be a mixture of species. If you provide a large number of specimens, the chances you provided specimens in good condition will increase. Also, depending upon the case and the species of the host will determine how detailed you might need the identification, i.e., to superfamily, genus, species.

Trematodes and Cestodes should be placed in a dish containing either tap water or physiological saline and allowed to relax for 30-60 minutes. They should then be fixed in AFA (85ml of 85% ethanol. 10ml commercial formalin, 5ml glacial acetic acid). Specimens may be stored and sent to the parasitologist in this solution. It is best to allow large tapeworms to flatten as much as possible and not pack them too tightly, as representative sections need to be mounted flat to see structures necessary for identification. Be sure you have included scoleces with tapeworms as these structures are often lost resulting in nonidentification in most cases. Flukes need to be flattened as well and this can be accomplished by fixing specimens between glass slides with small pressure applied to the upper slide.

Nematodes should be dipped in concentrated glacial acetic acid or hot 70% ethanol to fix them in as straight a posture as possible. After they have stopped writhing, transfer them into glycerin-alcohol (90ml 70% ethanol, 10ml glycerin). They may be stored indefinitely in this solution.

5) Epibiota collection:

<u>Supplied Materials:</u>
50 mL tubes for epibiota collection (3)

- Collect representative species and samples of epibiota from plastron, carapace, and skin.
- Place in 3 separate pre-labeled 50 mL tubes (one for each location: plastron, carapace, skin) with ethyl alcohol (preferred) or isopropyl alcohol and store at room temperature.
- Label tubes with date.
- Ship to Terry Norton at the end of the season.

6) Genetic sampling

1. Disinfect cutting board/surface with dilute bleach solution (1:10). Place a flipper on clean cutting board/surface.
2. Press the biopsy punch firmly into the flesh as close as possible to the posterior edge of the flipper and rotate one complete turn, cutting through the flesh of flipper. Alternatively use a sterile scalpel blade to remove a small plug of tissue.
3. Push out the tissue plug by inserting a wooden skewer or wire through the hollow end of the biopsy punch and place the tissue plug into a labeled (turtle number) vial with 95% EtOH.
4. Repeat 1-3 for an additional sample.
5. Do not reuse biopsy punch, wooden skewer or scalpel blade on another animal; disinfect wire skewer with bleach solution or another appropriate disinfectant if reusing.
6. Keep samples at room temperature and ship at the end of the season to Al Segars on frozen gel packs.

Persons performing necropsy: ____

Turtle ID#______________________________
(Stranding Reference)

Sea Turtle Necropsy Report

------------------------------------ EXTERNAL EXAM -----------------------------------

Ab=Abnormal NF=No Findings NE=Not Examined

Descriptions-include color, number, size, distribution, texture of lesions

Carapace Ab NF NE

Collect scute scraping

Trauma
Propeller wound Puncture wounds Missing scutes Bites Tumors
Description/additional comments:

Carapace Epibiota Ab NF NE

Collect representative samples of epibiota from carapace

Epibiota types
Sponges
Barnacle
Polychaetes

Goose barnacles
Leeches
Amphipod

Collect bone marrow from lateral scute – store in formalin, frozen, and make smears

Bryozoans
Other ___________________

Description/additional comments:

Plastron Ab NF NE

Trauma
Propeller wound Puncture wounds Missing scutes Bites Tumors

Plastron Marrow Ab NF NE

Description/additional comments:

Plastron Epibiota Ab NF NE

Collect representative samples of epibiota from plastron

Epibiota types
Sponges
Barnacle
Polychaetes

Goose barnacles
Leeches
Amphipod

Bryozoans
Other ___________________

Description/additional comments

Integument (Skin) **Ab** **NF** **NE**

Collect skin sample for genetics

Trauma

Sloughing
Necrosis
Net wounds
Fishing line/rope
Tumors

Propeller wounds
Other ___________________________

Region: **Head** **Neck** **Front Flippers** **Rear flippers** **Tail**

Description/additional comments:

Integument Epibiota **Ab** **NF** **NE**

Collect representative samples of epibiota from skin

Epibiota types
Sponges
Barnacle
Polychaetes

Goose barnacles
Leeches
Amphipod

Bryozoans
Other ___________________

Description/additional comments

Eyes **Ab** **NF** **NE**
Location Right Left Both
Description/additional comments:

Discharge Ab NF NE Location: Ocular Nasal Oral

For ocular: Right Left Bilateral
Color:____
Description/additional comments:

Cloaca Ab NF NE

Swollen Prolapsed Mucosal pseudomembrane
Feces color:_________________

Check for sex
cloaca beyond carapace
yes
no

Glans penis
yes
no

Description/additional comments:

Code 1 Only

MUSCULOSKELETAL SYSTEM

Skeleton and joints Ab NF NE
Joint/synovial fluid: Color______________

Characteristics:
Blood tinged
Cloudy/flocculent material
Plaques

Other__________
Viscosity:_______________

Fractures
No
Yes,
where? ____________________

Dislocation
No
Yes,
where? ____________________

Deformities
No
Yes,
where? ___________________

Description/additional comments:

Collect fat sample from left inguinal area

Musculature Ab NF NE

Characteristics:

Abcesses
Clotted blood
Pale

Gelatinized
Necrosis
Parasites
Cysts
Other (Specify) _________________________

Description/additional comments:

Coelomic cavity Ab NF NE

Fluid amount: _________ ml

Color:___________________ Viscosity:________________

Characteristics:

Clear
Cloudy/flocculent material
Blood tinged
Hemorrhage

Blood clots
Adhesions
Plaques
Gritty material (hard)

Peritoneum:

Characteristics:

Tumors
Abcesses /granulomas
Congested

Hemorrhage
Clotted blood

¨ Parasites (trematodes in mesenteric arteries)-SAVE__________Number

Description/additional comments:

CARDIOVASCULAR SYSTEM

Pericardial sac Ab NF NE
Fluid amount: _________ ml
Color:___________________ Viscosity:________________

Collect blood samples if the heart is still beating

Characteristics:

Clear
Cloudy/flocculent material
Blood tinged
Hemorrhage

Blood clots
Adhesions
Plaques
Caseous material

Description/additional comments:

Pulmonary arteries Ab NF NE & Aorta

Characteristics:

Thrombi
Plaques
Ruptures

Parasites (Trematodes--SAVE) ________ Number, ________ Size

Description/additional comments:

Atria Ab NF NE

Left Right Both

Characteristics:

Flaccid
Stiff
Thickened
Dilated

Hemorrhage
Pale areas
Parasites

Dimensions (cm (l) x cm (w) x cm (h)): ___________left, __________ right
Thickness of wall: right ______cm, left ______cm
Color: Surface:_______________________ Lumen:______________________
Description/additional comments:

Ventricle Ab NF NE
Characteristics:
Dimensions:_________________ cm (l) x cm (w) x cm (h)
Thickness of wall______________cm
Weight____________gm
Abscess/granulomas
Masses
Scars (fibrosis)
Friable

Color:___________________
Description/additional comments:

GASTROINTESTINAL SYSTEM

Liver Ab NF NE

Characteristics:
Dimensions:_________________ cm (l) x cm (w) x cm (h)
Weight: __________gm (or lbs/oz)
Color:____

Types of Lesions:
Abcesses/granulomas
Cysts
Masses

Congestion
Fibrosis
Necrosis

Collect 2 liver samples from right lobe with hexane-rinsed instruments #1 into foil; #2 into bag

Collect one liver sample (#3) with "No Teflon" instruments from left lobe into 50 mL tube

Severity of Lesions:
- Slight
- Mild
- Moderate
- Severe

Other:
- Fatty (greasy)
- Friable
- Cirrhotic
- Fractured

Parasites (SAVE): ____________Number

Description/additional comments:

Gall Bladder Ab NF NE

Bile: amount___________ml
Characteristics: thick thin
Color: serosa:_________mucosa:____________bile:__________
Diameter _________cm

South Carolina only: After liver samples are taken, collect bile with syringe and store in 50 mL tube

Types of Lesions:
- Abcesses/granulomas
- Cysts
- Masses

- Congestion
- Fibrosis
- Necrosis

Other:
- Friable
- Stones
- Gritty material

Parasites (SAVE): ____________Number
Description/additional comments:

Oral Cavity & Pharynx Ab NF NE

Characteristics:

- Ulcers
- Fluid
- Vomitus

Congestion
Parasites
Barnacles

Broken beak
Mandible
Maxilla

Foreign bodies ______________________________________

Description/additional comments:

Esophagus Ab NF NE

Characteristics:

Dilated
Constricted
Perforated

Fluid filled
Foreign bodies__________________________(SAVE)

Parasites (SAVE)_______________Number

Other ____

Mucosa:
Congested
Hemorrhagic
Ulcers

Necrosis
Thickened
Blunted papilla

Film on surface of mucosa

Color: mucosa:_____________________serosa:____________________
*Common in green sea turtles

Description/additional comments:

Stomach Ab NF NE

Characteristics:

Clotted blood
Thickened
Ruptures/laceration

Volvulus (twist)
Erosions

Ulcers:
Mild
Moderate
Severe

Focal
Multifocal
Focally-extensive

Extensive

Color: mucosa____________serosa:_____________contents:_____________

Contents:
Empty
Fluid
Dilated with gas

Mucus
Sand
Rocks

Other:__________________
Foreign bodies______________(SAVE)

Food:
Fish
Bivalves
Crustaceans

Cephalopods
Gastropods
Other________________

Undigested
Partially digested
Digested

Parasites:
Yes
No

<50
>50

Description/additional comments:

Mesentery Ab NF NE

Characteristics:

Hemorrhage
Clotted blood
Masses

Parasites (trematodes in mesenteric arteries)-SAVE______________Number

Description/additional comments:

Pancreas Ab NF NE
Characteristics:
Loss of lobulation Necrotic Edema Inflamed
Color: _______________________________________
Description/additional comments:

Small Intestine Ab NF NE

Characteristics:
Empty
Bile
Digesta

Other ___________
Foreign bodies______________(SAVE)

Parasites (SAVE): ____________Number

Color: mucosa____________serosa:_____________contents:_____________

Torsion/volvulus
Perforation
Masses
Abcesses

Constrictions
Diverticula
Ulcers (#)_____________

Pseudomembrane

Description/additional comments:

Colon Ab NF NE

Collect feces from colon and other noticeable parasites for parasitology

Characteristics:

Torsion/volvulus
Perforation
Masses
Abcesses

Constrictions
Empty
Feces

Fresh blood
Tarry
Other ___________________

Ulcers (#)_____________

Pseudomembrane

Parasites (SAVE): ____________Number

Color: mucosa____________serosa:_____________contents:_____________
Description/additional comments:

LYMPHOID SYSTEM

Spleen Ab NF NE

Characteristics: Enlarged Atrophied Friable
Dimensions: _________________ cm (l) x cm (w) x cm (h)
Weight: ___________gm

Abscess/granulomas
Masses
Scars (fibrosis)
Friable

Color:___________________________

Description/additional comments:

Thymus Ab NF NE
Characteristics: Enlarged Atrophied Friable
Color:____________________
Description/additional comments:

ENDOCRINE SYSTEM

Thyroid Ab NF NE

Characteristics: Normal Enlarged Atrophied Friable
Dimensions: ________________ cm (l) x cm (w) x cm (h)
Weight: ____________gm
Description/additional comments

Parathyroid(s) Ab NF NE

If unable to find, save lining over left thymus
Characteristics: Normal Enlarged Atrophied
Dimension(s): _________________ cm (l) x cm (w) x cm (h)
Weight: ____________gm
Description/additional comments:

Adrenal gland(s) Ab NF NE

Normal Enlarged Atrophied
Dimensions: _________________ cm (l) x cm (w) x cm (h)
Weight:___________gm

Description/additional comments:

RESPIRATORY SYSTEM

Trachea/Bronchi Ab NF NE

Abnormal Tissue:
  Trachea
  Bronchi

Characteristics:

Mucosa:
  White
  Vessels congested with blood

  Hemorrhage
  Ulcers

Trauma:
  Punctures
  Lacerations

Fluid:
  Serous
  Mucoid
  Purulent

Fluid or foam:
Color:____
  Parasites (SAVE): _________ Number
Description/additional comments:

Lungs Ab NF NE

Characteristics:

Color:

Lesion location:
  Left

Right
Both

Cranial
Caudal
Dorsal

Ventral
Middle

Distribution:
Diffuse
Focal
Multifocal

Severity
Mild
Moderate
Severe

Description/additional comments:

URINARY TRACT

Kidneys Ab NF NE
Characteristics:
Dimensions R_________________ cm (l) x cm (w) x cm (h)
Dimensions L_________________ cm (l) x cm (w) x cm (h)
Cortex color:____
Medulla color:____

Lesions:
Dialated with blood
Hemorrhage
Clotted blood

Abcesses/granulomas
Parasites
Cysts

Dialated with urine
Masses
Calculi

Congestion
Necrosis (focal, multifocal, diffuse)

Left weight__________gm____
Right weight_________gm____
Description/additional comments:

Ureters Ab NF NE
Characteristics:
Dilated Strictures Granulomas Calculi (SAVE)
Bilaterally symmetrical: Yes No
Description/additional comments:

Urethra Ab NF NE
Characteristics:
Patent: Yes No
Lesion: Ulcers Calculi Strictures
Description/additional comments:

Urinary bladder Ab NF NE

Characteristics:

Empty
Dilated
Thickened
Tumors

Color: mucosa:_______________urine:_________________

Mucosa:
Hemorrhagic
Ulcerated

Masses
Plaques
Necrotic

Urine: amount __________ml Consistency:____________________

Gritty material
Clear
Cloudy/flocculent material
Blood tinged

Parasites:
Yes(SAVE)

No

<5
5-20
>20

urinalysis strip (dip stick): ____

Description/additional comments:

REPRODUCTIVE

Gonads Ab NF NE

Characteristics:

Sex:
Male
Female

Maturity:
Mature
Immature

Enlarged
Involuted
Masses
Follicles

Necrotic

Description/additional comments:

Oviduct Ab NF NE
Characteristics:
¨ Enlarged ¨ Dilated with fluid ¨ Hemorrhagic ¨ Friable
¨ Tumors ¨ Masses ¨ Mucus ¨ Eggs
Description/additional comments:

NERVOUS SYSTEM

Section brain laterally.

Collect ½ brain with hexane-rinsed instruments into bag.

Collect other ½ into formalin for histopathology.

Dura mater and Inside calvarium Ab NF NE

Characteristics:
Hemorrhage Clotted blood Abcesses/Granulomas
Description/additional comments:

Central Nervous System Ab NF NE
Cerebral Spinal Fluid (CSF): __________ml color:___________________

Spinal Cord Fluid (SCF):__________ml color:___________________

Characteristics: (denote location of change in comments)

Distended red vessels
Abcesses/Granulomas
Clotted blood

Hemorrhage
Asymmetry
Edema
Black nodules

Lesions:
Brain
Spinal Cord ________(location)
Pituitary
Meninges

Weight: brain__________gm pituitary________gm

Description/additional comments:

Other:
Internal Auditory Meatus: Ab NF NE

Other information or description continuation page:

____

____

____

____

____

____

____

____

____

____

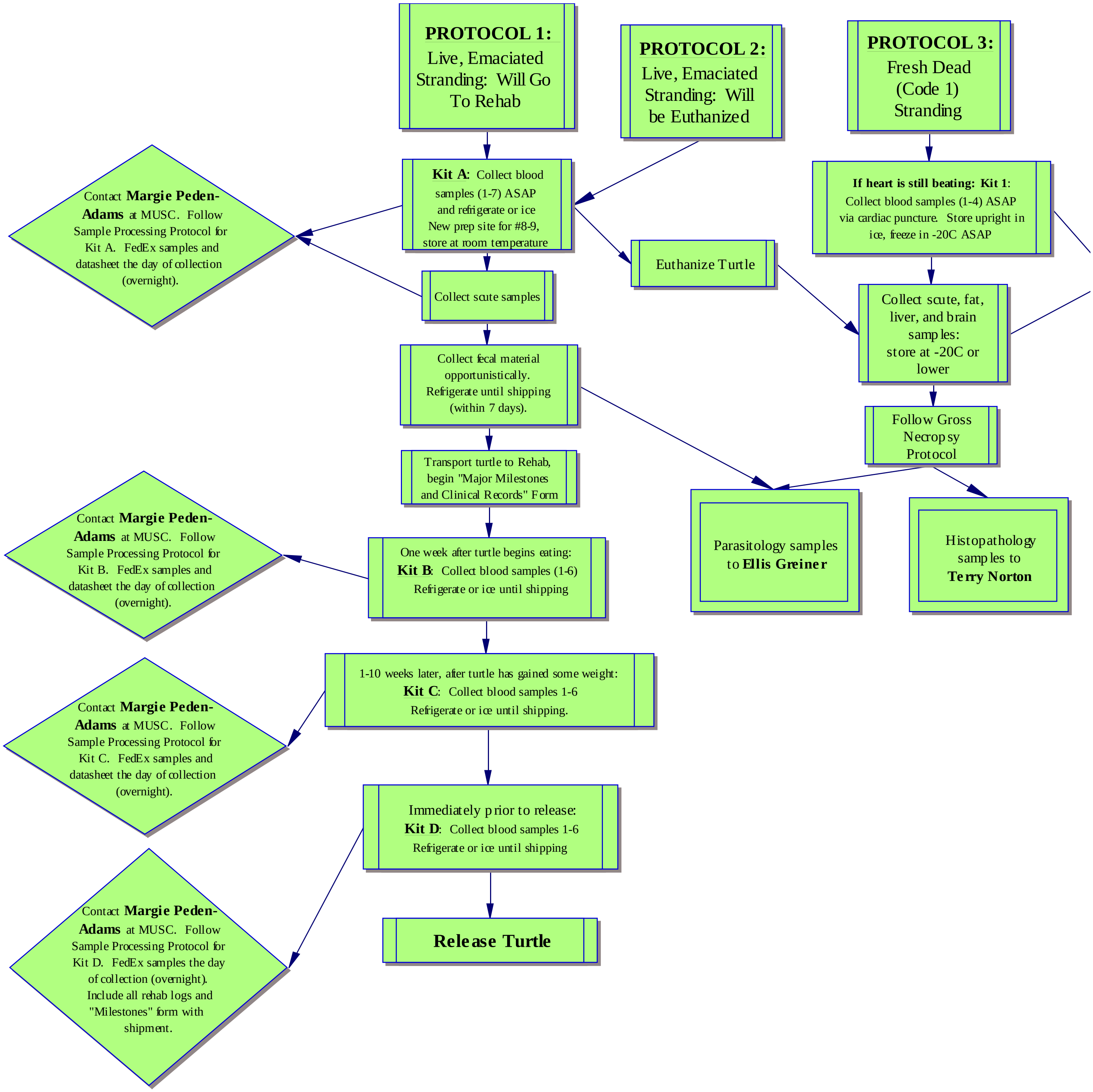

CONTACT INFORMATION:

CALL prior to or immediately after shipping. It is **critical** to notify researchers that these samples are in route.
Contact information was provided for each sample recipient here:

Blood samples must be shipped out on the same day as collection.

Samples must arrive within 24-36 hours after collection.

Please contact Margie Peden-Adams to make special arrangements if samples are collected Friday-Sunday.

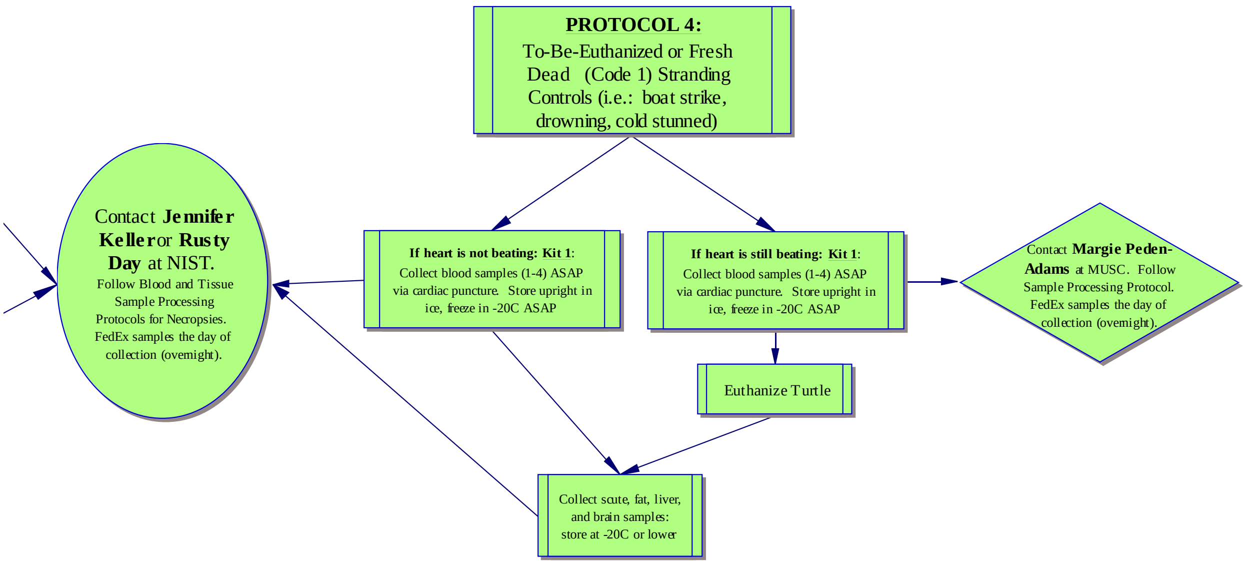
PROTOCOL 4:
To-Be-Euthanized or Fresh Dead (Code 1) Stranding Controls (i.e.: boat strike, drowning, cold stunned)
Contact Jennifer Keller or Rusty Day at NIST.
Follow Blood and Tissue Sample Processing Protocols for Necropsies. FedEx samples the day of collection (overnight).
If heart is not beating: Kit 1:
Collect blood samples (1-4) ASAP via cardiac puncture. Store upright in ice, freeze in -20C ASAP
If heart is still beating: Kit 1:
Collect blood samples (1-4) ASAP via cardiac puncture. Store upright in ice, freeze in -20C ASAP
Contact Margie Peden-Adams at MUSC. Follow Sample Processing Protocol. FedEx samples the day of collection (overnight).
Euthanize Turtle
Collect scute, fat, liver, and brain samples: store at -20C or lower

Protocol 1: Live, Emaciated Stranding--Will Go To Rehab
Sample Processing Protocol for Kit A

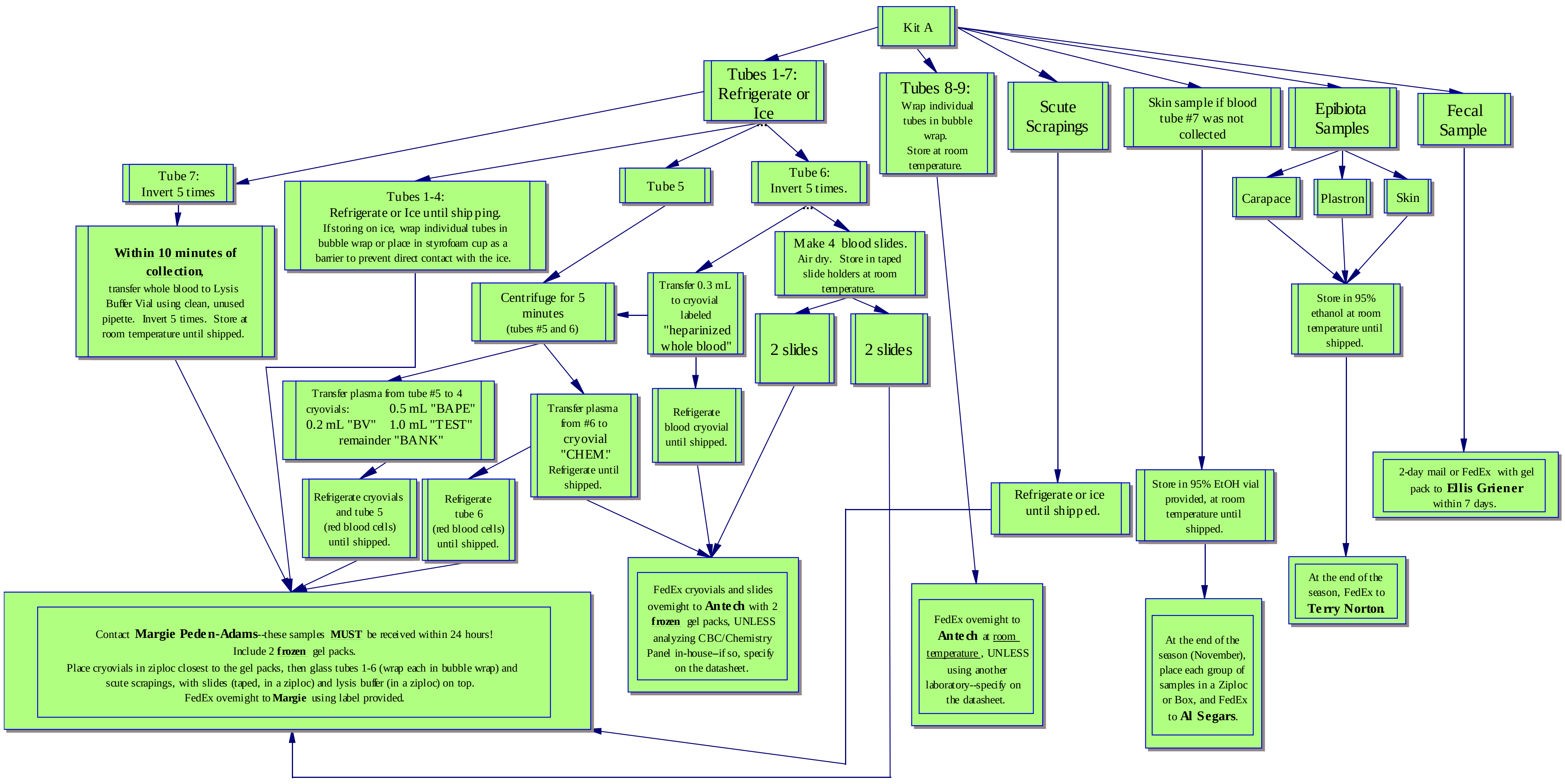

CONTACT INFORMATION:
CALL prior to or immediately after shipping. It is **critical** to notify researchers that these samples are in route.
Contact information was provided for each sample recipient here:

Protocol 1: Live, Emaciated Stranding Taken to Rehab
Contingency Plan
Turtle cannot withstand full sampling protocol

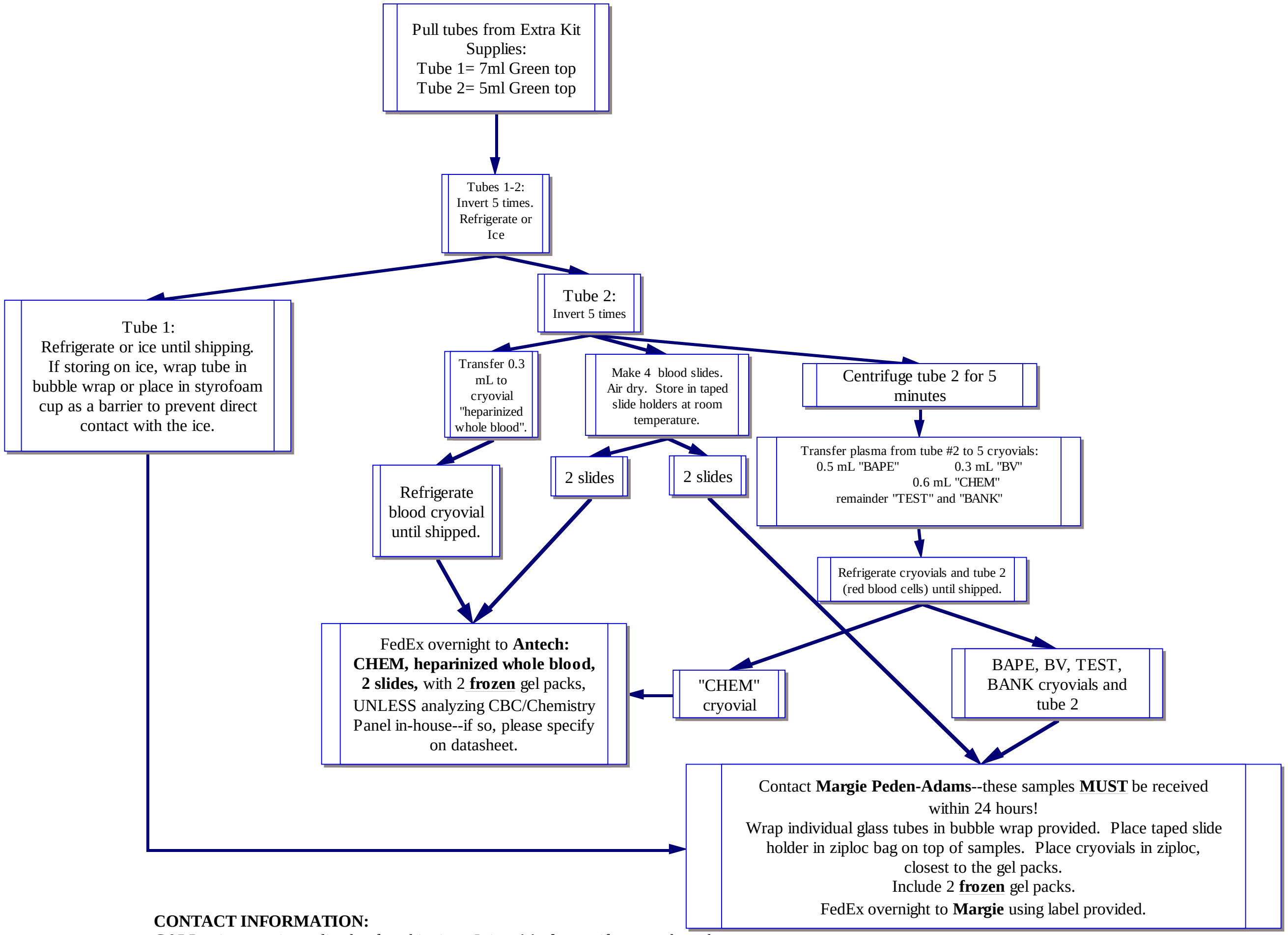

CONTACT INFORMATION:
CALL prior to or immediately after shipping. It is **critical** to notify researchers that these samples are in route.
Contact information was provided for each sample recipient here:

Packing for Margie's Shipment

Slides (taped closed, in ziploc)
Tubes 1-2 (bubble-wrapped)
Cryovials BAPE, BV, TEST, BANK (in ziploc)
Two Gel Packs

Protocol 1: Live, Emaciated Stranding Taken to Rehab
Sample Processing for Kits B and C

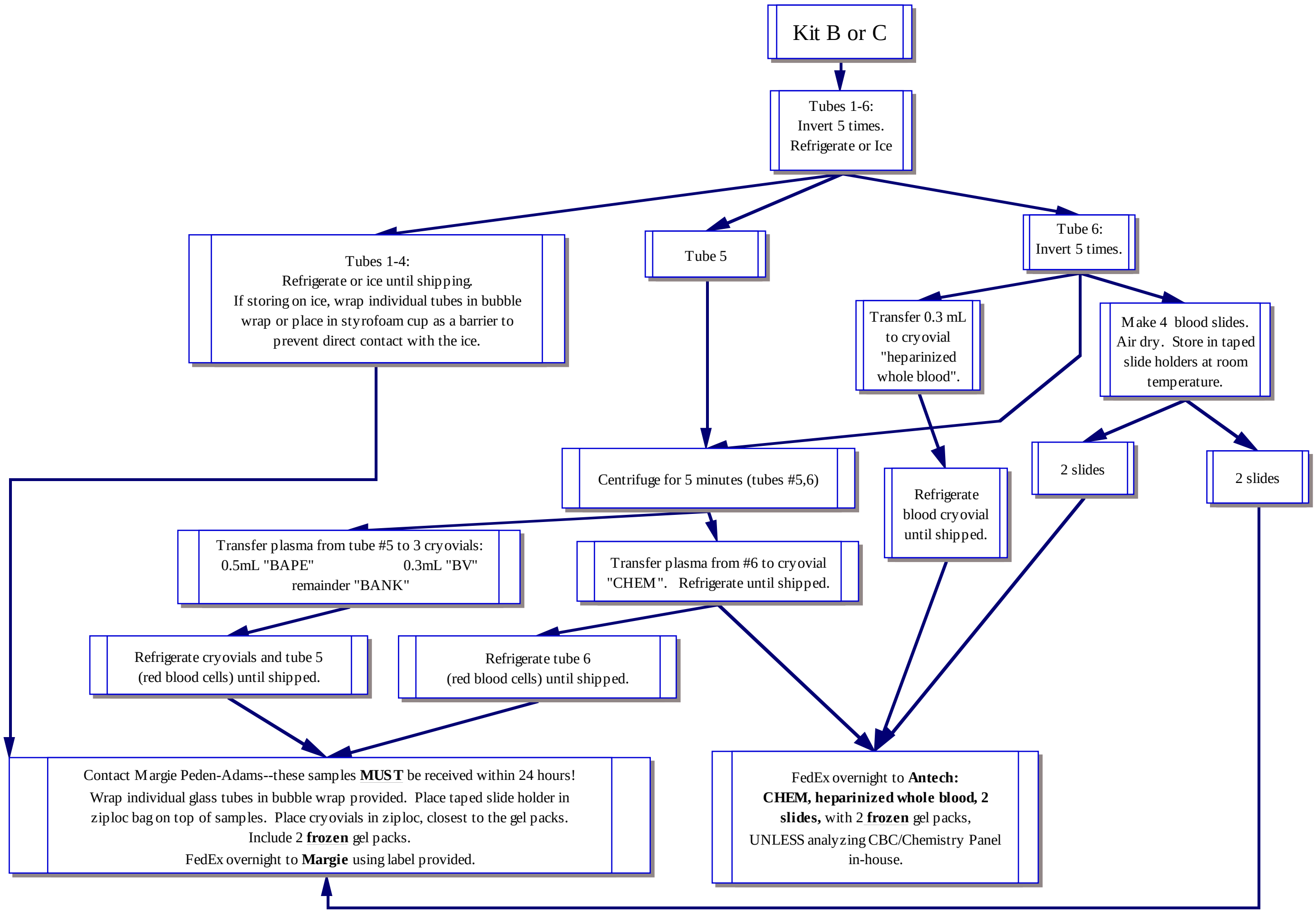

CONTACT INFORMATION:
CALL prior to or immediately after shipping. It is **critical** to notify researchers that these samples are in route.
Contact information was provided for each sample recipient here:

Packing for Margie's Shipment

Slides (taped closed, in ziploc)
Tubes 1-6 (bubble-wrapped)
Cryovials BAPE, BV, BANK (in ziploc)
Two Gel Packs

Protocol 1: Live, Emaciated Stranding Taken to Rehab

Sample Processing for Kits B and C

Kit B or C

I. **Tubes 1-6:**

   Invert 5 times.

Refrigerate or Ice

A. Tube 6:

Invert 5 times.

1. **Transfer 0.3 mL to cryovial "heparinized whole blood".**
   a. **Refrigerate blood cryovial until shipped.**
      (1) **FedEx overnight to Antech: CHEM, heparinized whole blood, 2 slides, with 2 <u>frozen</u> gel packs, UNLESS analyzing CBC/Chemistry Panel in-house.**
2. **Make 4 blood slides. Air dry. Store in taped slide holders at room temperature.**
   a. **2 slides**
   b. **2 slides**
      (1) **Contact Margie Peden-Adams--these samples <u>MUST</u> be received within 24 hours!**
      Wrap individual glass tubes in bubble wrap provided. Place taped slide holder in ziploc bag on top of samples. Place cryovials in ziploc, closest to the gel packs.

   Include 2 <u>frozen</u> gel packs.

   FedEx overnight to Margie using label provided.
3. **Centrifuge for 5 minutes (tubes #5,6)**
   a. **Transfer plasma from tube #5 to 3 cryovials:**
      0.5mL "BAPE" 0.3mL "BV"
      remainder "BANK"

      (1) **Refrigerate cryovials and tube 5 (red blood cells) until shipped.**
   b. **Transfer plasma from #6 to cryovial "CHEM". Refrigerate until shipped.**
      (1) **Refrigerate tube 6 (red blood cells) until shipped.**

B. Tube 5

C. Tubes 1-4:

Refrigerate or ice until shipping.

If storing on ice, wrap individual tubes in bubble wrap or place in styrofoam cup as a

barrier to prevent direct contact with the ice.

Slides (taped closed, in ziploc)

Tubes 1-6 (bubble-wrapped)

Cryovials BAPE, BV, BANK (in ziploc)

Two Gel Packs

Packing for Margie's Shipment

Protocol 1: Live, Emaciated Stranding Taken to Rehab
Sample Processing for Kit D

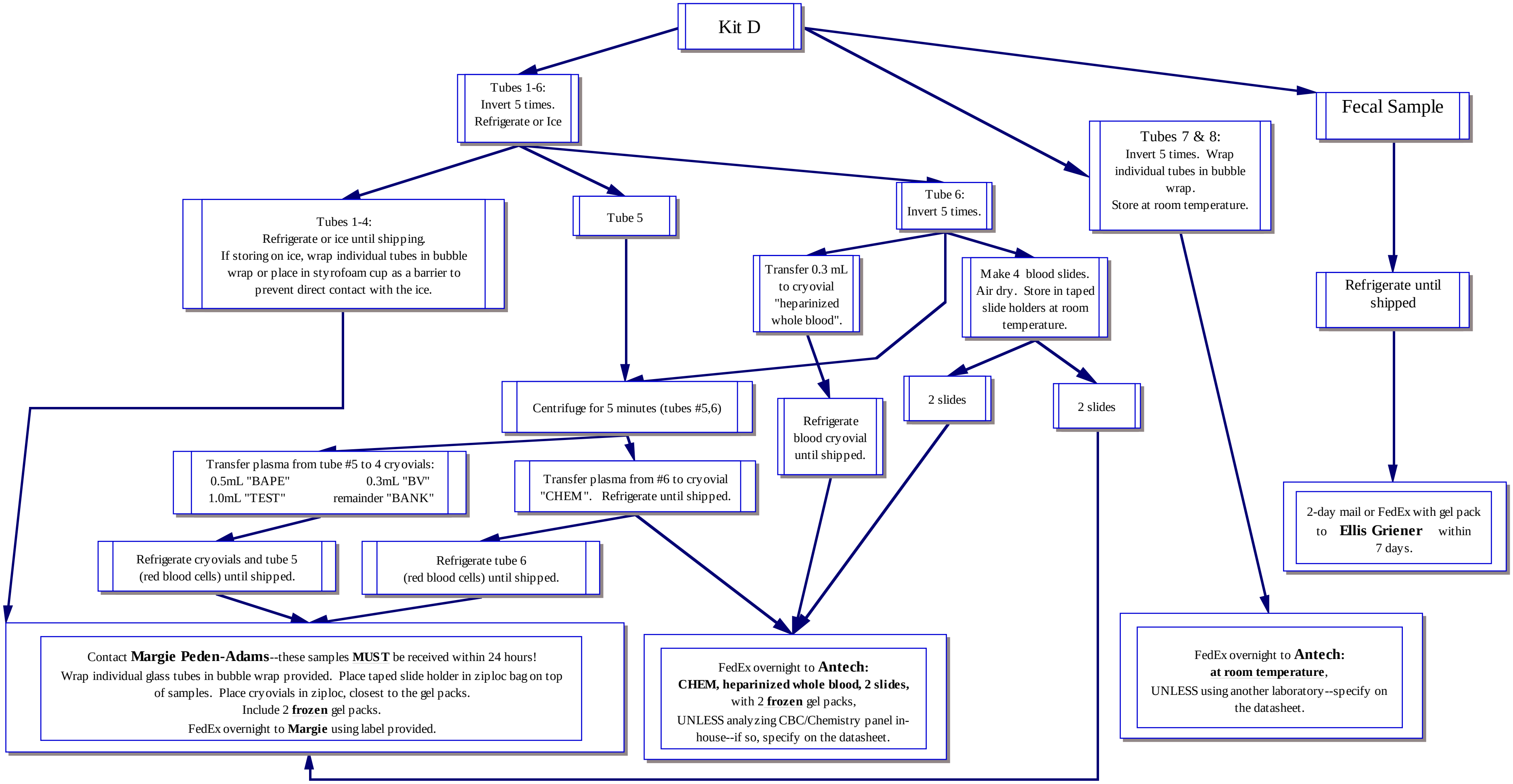

CONTACT INFORMATION:
CALL prior to or immediately after shipping. It is **critical** to notify researchers that these samples are in route.
Contact information was provided for each sample recipient here:

Packing for Margie's Shipment

Slides (taped closed, in ziploc)
Tubes 1-6 (bubble-wrapped)
Cryovials BAPE, BV, TEST, BANK (in ziploc)
Two Gel Packs

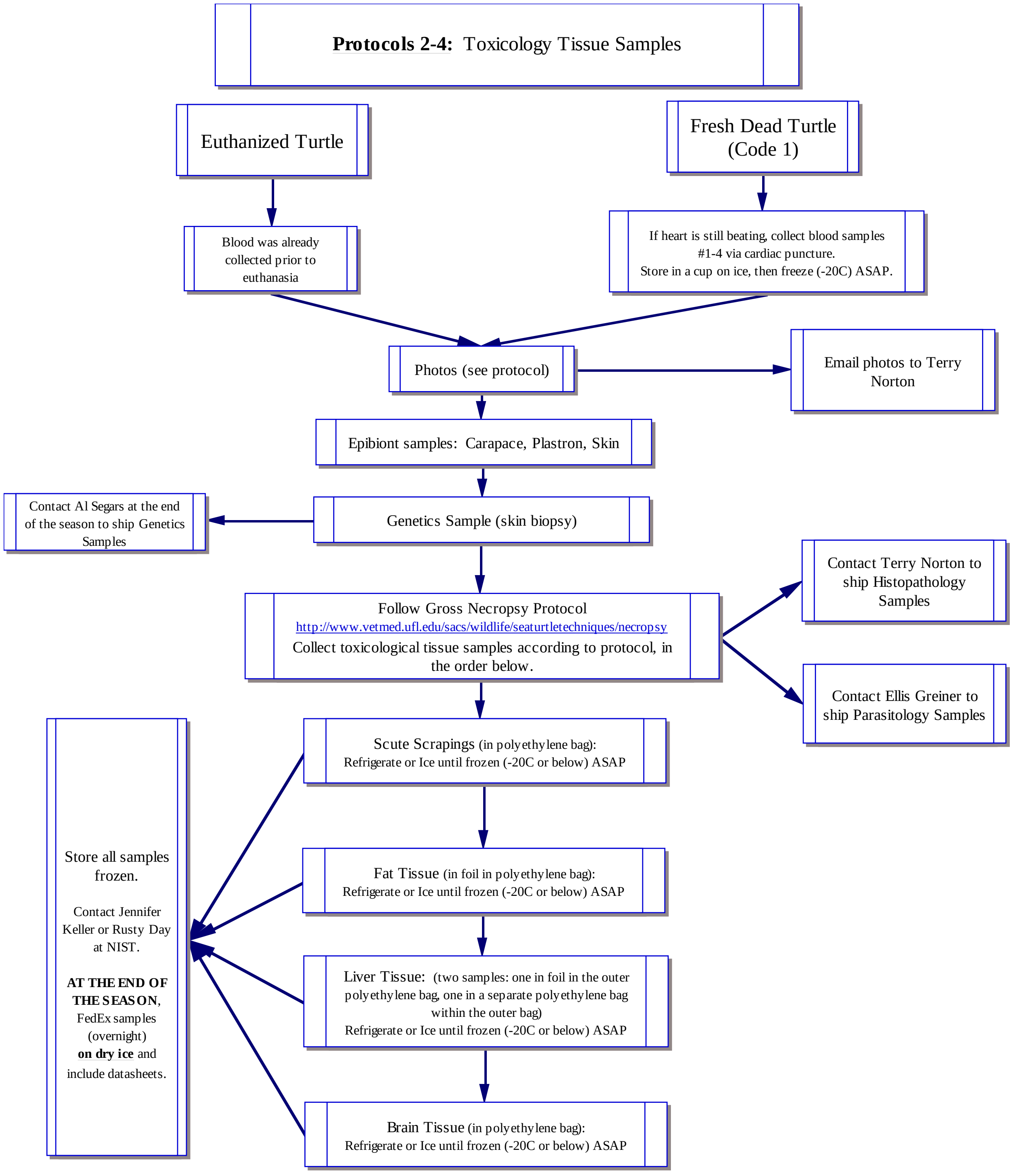

CONTACT INFORMATION:
CALL prior to or immediately after shipping. It is **critical** to notify researchers that these samples are in route.
Contact information was provided for each sample recipient here.

<u>Protocols 2-4:</u> **Toxicology Tissue Samples**

Euthanized Turtle

I. **Blood was already collected prior to euthanasia**

A. **Photos (see protocol)**

1. **Epibiont samples: Carapace, Plastron, Skin**

a. **Genetics Sample (skin biopsy)**

(1) **Follow Gross Necropsy Protocol**

http://www.vetmed.ufl.edu/sacs/wildlife/seaturtletechniques/necropsy

Collect toxicological tissue samples according to protocol, in the order below.

(a) **Scute Scrapings (in polyethylene bag):**

Refrigerate or Ice until frozen (-20C or below) ASAP

i) **Fat Tissue (in foil in polyethylene bag):**

Refrigerate or Ice until frozen (-20C or below) ASAP

(1) **Liver Tissue: (two samples: one in foil in the outer polyethylene bag, one in a separate polyethylene bag within the outer bag)**

Refrigerate or Ice until frozen (-20C or below) ASAP

(a) **Brain Tissue (in polyethylene bag):**

Refrigerate or Ice until frozen (-20C or below) ASAP

ii)

Store all samples frozen.

Contact Jennifer Keller or Rusty Day at NIST.

AT THE END OF THE SEASON,

FedEx samples (overnight) <u>on dry ice</u> and include datasheets.

(b) **Contact Terry Norton to ship Histopathology Samples**

(c) Contact Ellis Greiner to ship Parasitology Samples

(2) Contact Al Segars at the end of the season to ship Genetics Samples

2. Email photos to Terry Norton

Fresh Dead Turtle (Code 1)

I. If heart is still beating, collect blood samples #1-4 via cardiac puncture.
Store in a cup on ice, then freeze (-20C) ASAP.

Debilitated Loggerhead Study

Datasheet for PROTOCOL 1: Turtles sent to rehabilitation

Major Milestones and Clinical Records

Turtle Stranding ID (given by State): ______________________ State: ________

Rehabilitation Center Name: ______________________

Rehabilitation Center's Name for the turtle: ______________________

Milestone	Date/location	Time	by Whom
Turtle discovered			
Turtle arrived at rehab			
Turtle began eating			
Turtle condition changed			
How?			
Turtle recovered completely			
Turtle released			

Record of measurements

Date	SCL* cm or in	Weight kg or lbs	Depth cm or in	Plastron concavity measurement* cm or in

Record of samples

Date	Kit number

SCL = straight carapace length from nuchal notch to posterior marginal notch

*See diagram in protocol for how to take this measurement

Record of notable changes in condition or behavior

Date	Health measures (attach additional lab data)			Description of change in condition or behavior
	PCV	TS	glucose	

PCV = packed cell volume or hematocrit

TS= total solids via refractometer

Keep datasheet with turtle's records until released, then send copy to Margie Peden-Adams with the FINAL blood samples

Debilitated Loggerhead Study

Datasheet for PROTOCOL 1: Turtles sent to rehabilitation

Major Milestones and Clinical Records

Treatment descriptions

Drug treatments (type, amount, frequency administered, dates)

Date	Drug	Strength (dose)	Frequency Administered	Route of delivery

Feeding schedules (food items, amount, frequency provided)

Date	Amount	Food Item(s)	This diet was fed for how long and how often?

Keep datasheet with turtle's records until released, then send copy to Margie Peden-Adams with the FINAL blood samples

Debilitated Loggerhead Study

Datasheet for PROTOCOL 1: Turtles sent to rehabilitation

Initial Sampling--Kit A

Turtle Stranding ID (given by State): ____________________ State: ________

Sampling kit number: ____________________

Rehabilitation Center Name: ____________________

Rehabilitation Center's Name for the turtle: ____________________

**Timing of Major Events**	Date/ location	Time	by whom
Turtle discovered			
Blood samples taken			
Blood samples refrigerated			
Blood samples shipped out			
Blood samples arrived in Charleston			
Blood samples processed in Charleston			
Blood samples banked in Charleston			
Scute scrapings taken			
Scute scrapings frozen in Charleston			
Turtle arrived at rehab center			

Initial measurements

Photos taken (page 4 of protocol)? yes no

Body condition (circle one) good fair poor

Straight carapace length from nuchal notch to most posterior notch ____________ cm or in

Straight carapace length from nuchal notch to most posterior tip ____________ cm or in

Straight carapace width ____________ cm or in

Body depth ____________ cm or in

Plastron concavity measurement (see protocol for method) ____________ cm or in

Weight ____________ kg or lbs

Date measurements taken ____________

Notes/ Checklist on Blood sampling

Were tubes collected in the proper order (shown below)? yes no

If no, how were they collected?

Were all tubes filled to capacity? Provide estimated volumes and tube lot numbers here:

Tube	Estimated mL	Lot #	Purpose
7mL Green-top tube #1:			immune function
5mL Green-top tube #2:			metals analysis
5mL Green-top tube #3:			OC analysis
5mL Green-top tube #4:			OC analysis
5mL Green-top tube #5:			bile acids,biliverdin,electrophoresis,testosterone,bank
5mL Green-top tube #6:			CBC, Blood chemistry
3mL Red-top tube #7:			genetics- need 0.5- 1 ml
			(postpone tube 7 if severely compromised)
Culture tube # 8*			*** new prep/ needle**

Send datasheet with blood, cryovials, "Al Segars" slides and scute samples to Margie Peden-Adams.

Debilitated Loggerhead Study

Datasheet for PROTOCOL 1: Turtles sent to rehabilitation

Initial Sampling--Kit A

SAMPLING KIT NUMBER:

Blood slides (4) made? yes no

Was more than one needle used to collect tubes(1-4)? yes no

If so, which tube received the new needle?

New needle/ skin prep for tube 8? yes no

Was the turtle transported before blood was taken? yes no

If so, how and duration of trip?

Notes on Scute Scrapings or General Comments:

Additional sampling:

	Date collected	Date shipped	Rx/dose/date
Fecal Sample			
	Date collected	Date shipped	
Epibiota Samples			
	Date	Biopsy site	Lab sent to
Biopsy taken (lesion or mass)			
Biopsy taken for genetics (if no tube #7)			send to Al Segars

Blood Processing Checklist:

3mL Red-top tube #7:

sample transferred to lysis buffer

5mL Green-top tube #5:

Cryovial(s)	Volume
BAPE	
BV	
TEST	
BANK	

More than 1 BANK cryovial used? Yes No

If Yes, how many, and volumes in each:

5mL Green-top tube #6:

Cryovial(s)	Volume
CHEM	
Heparinized blood	

If you used your own in-house lab, explain your processing methods and intrumentation used:

Did you retain and ship red blood cells from tubes #5 and 6? yes no

Send datasheet with blood, cryovials, "Al Segars" slides and scute samples to Margie Peden-Adams.

Debilitated Loggerhead Study

Datasheet for PROTOCOL 1: Turtles sent to rehabilitation

Second Sampling--Kit B

(1 week after turtle begins eating)

Turtle Stranding ID (given by State): ______________________ State: __________

Sampling kit number: ______________________

Rehabilitation Center Name: ______________________

Rehabilitation Center's Name for the turtle: ______________________

**Timing of Major Events**	Date	Time	by whom
Turtle began feeding			
Blood samples taken			
Blood samples refrigerated			
Blood samples shipped out			
Blood samples arrived in Charleston			
Blood samples processed in Charleston			
Blood samples banked in Charleston			

Body condition (circle one) good fair poor

Measurements

Straight carapace length from nuchal notch to most posterior notch __________ cm or in

Body depth __________ cm or in

Plastron concavity measurement (see protocol for method) __________ cm or in

Weight __________ kg or lbs

Date measurements taken __________

Notes/Checklist on Blood sampling

Were tubes collected in the proper order (shown below)? yes no

If no, how were they collected?

Were all tubes filled to capacity? Provide estimated volumes and tube lot numbers here:

Tube	Estimated mL	Lot #	Purpose
7mL Green-top tube #1:			immune function
5mL Green-top tube #2:			metals
5mL Green-top tube #3:			OC analysis
5mL Green-top tube #4:			OC analysis
5mL Green-top tube #5:			bile acids, biliverdin, electrophoresis,bank
5mL Green-top tube #6:			CBC, Blood chem

Blood slides (4) made? yes no

Was more than one needle used to collect these tubes? yes no

If so, which tube received the new needle?

Send datasheet with blood samples to Margie Peden-Adams

Debilitated Loggerhead Study

Datasheet for PROTOCOL 1: Turtles sent to rehabilitation

Second Sampling--Kit B

(1 week after turtle begins eating)

Notes on Turtle's condition

Provide observations of changes in feeding, swimming, and diving behaviors, weight gain, changes in skin coloration, and attach recent blood chemistry values with dates (if available)

Datasheet for PROTOCOL 1: Turtles sent to rehabilitation

Third Sampling--Kit C

(within 10 weeks of second sample and after slight improvement, but not fully recovered)

Turtle Stranding ID (given by State): ______________________ State: __________

Sampling kit number: ______________________

Rehabilitation Center Name: ______________________

Rehabilitation Center's Name for the turtle: ______________________

**Timing of Major Events**	Date	Time	by whom
Turtle showed observable improvement			
Describe improvement:			
Blood samples taken			
Blood samples refrigerated			
Blood samples shipped out			
Blood samples arrived in Charleston			
Blood samples processed in Charleston			
Blood samples banked in Charleston			

Body condition (circle one) good fair poor

Measurements

Straight carapace length from nuchal notch to most posterior notch ______________ cm or in

Body depth ______________ cm or in

Plastron concavity measurement (see protocol for method) ______________ cm or in

Weight ______________ kg or lbs

Date measurements taken ______________

Notes/Checklist on Blood sampling

Were tubes collected in the proper order (shown below)? yes no

If no, how were they collected?

Were all tubes filled to capacity? Provide estimated volumes and tube lot numbers here:

Tube	Estimated mL	Lot #	Purpose
7mL Green-top tube #1:			immune function
5mL Green-top tube #2:			metals
5mL Green-top tube #3:			OC analysis
5mL Green-top tube #4:			OC analysis
5mL Green-top tube #5:			bile acids, biliverdin, electrophoresis,bank
5mL Green-top tube #6:			CBC, Blood chem

Blood slides (4) made? yes no

Was more than one needle used to collect these tubes? yes no

If so, which tube received the new needle?

Send datasheet with blood samples to Margie Peden-Adams

Datasheet for PROTOCOL 1: Turtles sent to rehabilitation

Third Sampling--Kit C

(within 10 weeks of second sample and after slight improvement, but not fully recovered)

Notes on Turtle's condition

Provide observations of changes in feeding, swimming, and diving behaviors, weight gain, changes in skin coloration, and attach recent blood chemistry values with dates (if available)

Datasheet for PROTOCOL 1: Turtles sent to rehabilitation

Final Recovered Sampling--Kit D

(fully recovered, just prior to release)

Turtle Stranding ID (given by State): ______________ State: ______________

Sampling kit number: ______________

Rehabilitation Center Name: ______________

Rehabilitation Center's Name for the turtle: ______________

Tags Prior to Release (fill in only if tags are known - provide tag numbers later if need be)

Tag #	Tag Type (i.e. PIT or flipper tag)	Tag Location (i.e. front right flipper)

**Timing of Major Events**	Date	Time	by whom
Turtle showed complete recovery			
Blood samples taken			
Blood samples refrigerated			
Blood samples shipped out			
Blood samples arrived in Charleston			
Blood samples processed in Charleston			
Blood samples banked in Charleston			

Body condition (circle one) good fair poor

	date collected	date shipped	Rx/dose/date
Fecal Sample			

Final Measurements

Straight line (using calipers): **cm or in**

Carapace length: Notch-Notch ______________ Notch-Tip ______________

Carapace width (widest point) ______________

Body depth (maximum) ______________

Head width ______________

Plastron concavity (see protocol for method) ______________

Plastron length (midline, including small scale) ______________

Curved: **cm or in**

Carapace length: Notch-Notch ______________ Notch-Tip ______________

Carapace width (widest point) ______________

Plastron width (widest point) ______________

Tail length: To Vent ______________ To Tip ______________

Weight: ______________ **kg or lbs**

Date measurements taken: ______________

Send this datasheet, the "Milestones" datasheet, and clinical data if available with blood samples to Margie Peden-Adams

Datasheet for PROTOCOL 1: Turtles sent to rehabilitation

Final Recovered Sampling--Kit D

(fully recovered, just prior to release)

Notes/Checklist on Blood sampling

Were tubes collected in the proper order (shown below)? yes no
If no, how were they collected?

Were all tubes filled to capacity? Provide estimated volumes and tube lot numbers here:

Tube	Estimated mL	Lot #	Purpose
7mL Green-top tube #1:			immune function
5mL Green-top tube #2:			metals analysis
5mL Green-top tube #3:			OC analysis
5mL Green-top tube #4:			OC analysis
5mL Green-top tube #5:			bile acids,biliverdin,electrophoresis,testosterone,bank
5mL Green-top tube #6:			CBC, Blood chemistry
Culture tube # 8*			*** new prep/ needle**

Blood slides (4) made? yes no

Was more than one needle used to collect tubes(1-4)? yes no
If so, which tube received the new needle?
New needle/ skin prep for tube 8? yes no
Was the turtle transported before blood was taken? yes no
If so, how and duration of trip?

Notes on Turtle's condition

Provide observations of changes in feeding, swimming, and diving behaviors, weight gain, changes in skin coloration, and attach recent blood chemistry values with dates (if available)

Send this datasheet, the "Milestones" datasheet, and clinical data if available with blood samples to Margie Peden-Adams

Datasheet for PROTOCOL 2: Turtles to be euthanized
Blood Sampling

Turtle Stranding ID (given by State): ______________________ State: __________

Sampling kit number: ______________________

**Timing of Major Events**	Date	Time	by whom
Turtle discovered			
Blood samples taken			
Blood samples refrigerated			
Blood samples shipped out			
Blood samples arrived in Charleston			
Blood samples processed in Charleston			
Blood samples banked in Charleston			

	Date collected	Date shipped
Fecal Sample, if available		

Turtle Measurements

Straight carapace length from nuchal notch to most posterior notch __________ cm or in

Body depth __________ cm or in

Plastron concavity measurement (see protocol for method) __________ cm or in

Weight __________ kg or lbs

Date measurements taken __________

Notes/Checklist on Blood sampling

Were tubes collected in the proper order (shown below)? yes no

If no, how were they collected?

Were all tubes filled to capacity? Provide estimated volumes and tube lot numbers here:

Tube	Estimated mL	Lot #	Purpose
5mL Green-top tube #1:			OC analysis
5mL Green-top tube #2:			OC analysis
5mL Green-top tube #3:			metals analysis
5mL Green-top tube #4:			metals analysis

Was more than one needle used to collect tubes 1-4? yes no
If so, which tube received the new needle?
Was the turtle transported before blood was taken? yes no

Euthanasia

Date	Type/ volume of euthanasia solution	Euthanized by:

Send datasheet with blood samples to Jennifer Keller.

Datasheet for PROTOCOL 3: Necropsies

Euthanized turtle

Turtle Stranding ID (given by State): ____________________ State: __________

Timing of Major Events	Date	Time	by whom
Turtle discovered			
Turtle euthanized			
Turtle iced/frozen at _________ ^{o}C			
Necropsy begins			
Scute scraping taken			
Fat sample taken			
3 Liver samples taken			
Brain sample taken			
Samples iced or refrigerated			
Samples frozen at _________ ^{o}C			
Samples shipped			
Tissue samples banked in Charleston			
Internal parasite samples taken			
Epibiota samples taken			
Genetics sample taken			

Storage locations of samples prior to shipping to NIST

	Institution	Freezer name	^{o}C	Container description	Contact person	Email or Phone
Scute						
Fat						
3 Liver						
Brain						

Fill out below only if blood was not taken before the turtle was euthanized.

Turtle Measurements

Straight carapace length from nuchal notch to most posterior notch ____________ cm or in

Body depth ____________ cm or in

Plastron concavity measurement (see protocol for method) ____________ cm or in

Weight ____________ kg or lbs

Date measurements taken ____________

Euthanasia

Date	Type/ volume of euthanasia solution	Euthanized by:

Fax datasheet to Jennifer Keller (843-762-8742) and send original and any other necropsy report with the frozen samples

Datasheet for PROTOCOL 3: Necropsies
Dead Stranding

Turtle Stranding ID (given by State): ____________________ State: ________

Sampling kit number: ____________________

Timing of Major Events	Date	Time	by whom
Turtle discovered live or dead			
Turtle died natural or euthanized			
Turtle iced/frozen at _________ ^{o}C			
Necropsy begins			
Scute scraping taken			
Fat sample taken			
3 Liver samples taken			
Blood samples taken neck or heart			
Fecal samples taken			
Brain sample taken			
Samples iced or refrigerated			
Samples frozen at _________ ^{o}C			
Samples shipped			
Tissue samples banked in Charleston			
Internal parasite samples taken			
Epibiota samples taken			
Genetics sample taken blood or skin			

Turtle Measurements

Photos taken (page 4 of protocol)? yes no

Body condition (circle one) good fair poor

Straight carapace length from nuchal notch to most posterior notch ____________ cm or in

Straight carapace length from nuchal notch to most posterior tip ____________ cm or in

Straight carapace width ____________ cm or in

Body depth ____________ cm or in

Plastron concavity measurement (see protocol for method) ____________ cm or in

Weight ____________ kg or lbs

Date measurements taken ____________

Notes on Blood sampling

Was the heart still beating? yes no

Were tubes collected in the proper order (shown below)? yes no

If no, how were they collected?

Were all tubes filled to capacity? Estimated volumes: Lot #:

5mL Green-top tube #1: ____________________

5mL Green-top tube #2: ____________________

5mL Green-top tube #3: ____________________

5mL Green-top tube #4: ____________________

Fax datasheet to Jennifer Keller (843-762-8742) and send original and any other necropsy report with the frozen samples

Datasheet for PROTOCOL 3: Necropsies

Dead Stranding

Turtle Stranding ID (given by State): ______________________ State: ________

Sampling kit number: ______________________

Provide a full description of how the blood samples were taken:

Notes on Tissue sampling

Were proper provided instruments used on all samples?
If no, please describe.

Did any tissues touch another surface other than provided instruments and storage materials?
If yes, please describe.

Scute samples:

Fat Sample Description:
color (i.e. yellow, green, brown, black)
texture (i.e. buttery, watery, flaccid)
sampling location if other than the left inguinal region

Liver Sample: Was gallbladder broken before sampling? yes no
If yes, did bile come in contact with these particular samples? yes don't know no

Brain Sample: Was brain sampled in whole or only a portion?

Storage locations of samples prior to shipping to NIST

	Institution	Freezer name	^{o}C	Container description	Contact person	Email or Phone
Scute						
Fat						
3 Liver						
Blood						
Brain						

Datasheet for PROTOCOL 3: Necropsies

Dead Stranding

Turtle Stranding ID (given by State): ______________________ State: ________

Sampling kit number: ______________________

Blood processing

Tube # Process

1

2

3

4

Fecal sample description date shipped ________________

Parasites collected

Tissue origin Parasite description Processing date shipped ________________

Tissue checklist - Collect tissues that are not 'greyed' out.

Tissue	Histopathology	Frozen for virus	Toxicology	Other
Epibiota - carapace				
Epibiota - skin				
Epibiota - plastron				
Scute scrapings				
Blood - neck				genetics
Skin				genetics
Fat				
Muscle				
Heart				
Blood - heart				
Spleen				
Liver			(3)	
Gall bladder				
Bile				Tom S.
Esophagus				
Stomach				
Small intestine				
Pancreas				
Colon				
Kidney				
Bladder				
Gonad				
Trachea				
Bronchi				
Lungs				
Adrenal				
Brain				
Bone marrow	(formalin&slide)		Color of marrow ________________	
